# Cell-type expression and activation by light of neuropsins in the developing and mature *Xenopus* retina

**DOI:** 10.3389/fncel.2023.1266945

**Published:** 2023-09-20

**Authors:** Lawrence L. H. Man, Samuel S. Storey, Gabriel E. Bertolesi, Sarah McFarlane

**Affiliations:** Department of Cell Biology and Anatomy, Hotchkiss Brain Institute, Alberta Children’s Hospital Research Institute, University of Calgary, Calgary, AB, Canada

**Keywords:** retina, opsin, eye, neuronal circuit, frog, evolution, development

## Abstract

Photosensitive opsins detect light and perform image- or nonimage-forming tasks. Opsins such as the “classical” visual opsins and melanopsin are well studied. However, the retinal expression and functions of a novel family of neuropsins are poorly understood. We explored the developmental time-course and cell-type specificity of neuropsin (*opn5*, *6a*, *6b*, and *8*) expression in *Xenopus laevis* by *in situ* hybridization and immunohistochemistry. We compared the *Xenopus* results with publicly available single cell RNA sequencing (scRNA-seq) data from zebrafish, chicken, and mouse. Additionally, we analyzed light-activation of neuropsin-expressing cells through induction of *c-fos* mRNA. *opn5* and *opn8* expression begins at stage 37/38 when the retinal circuits begin to be activated. Once retinal circuits connect to the brain, *opn5* mRNA is distributed across multiple retinal cell types, including bipolar (~70%–75%), amacrine (~10%), and retinal ganglion (~20%) cells, with *opn8* present in amacrine (~70%) and retinal ganglion (~30%) cells. *opn6a* and *opn6b* mRNAs emerge in newborn-photoreceptors (stage 35), and are colocalized in rods and cones by stage 37/38. Interestingly, in the mature larval retina (stage 43/44), *opn6a* and *opn6b* mRNAs become preferentially localized to rods and cones, respectively, while newborn photoreceptors bordering the proliferative ciliary marginal zone express both genes. In zebrafish, *opn6a* and *opn6b* are also expressed in photoreceptors, while Müller glia and amacrine cells express *opn8c*. Most neuropsin-expressing retinal ganglion cells display *c-fos* expression in response to light, as do over half of the neuropsin-expressing interneurons. This study gave a better understanding of retinal neuropsin-expressing cells, their developmental onset, and light activation.

## Introduction

Opsins are photosensitive proteins that capture and decode light information for visual and non-visual tasks. In the retina, the function and physiology of classical opsins [rhodopsin (RHO) and cone-opsins] and melanopsin (*opn4*) are well known (Thoreson and Dacey, 2019). Less understood, however, is the physiological role of the novel neuropsin family (*opn5*, *6*, *7*, *8*, and *9*). *opn5* is well studied as the only neuropsin conserved in the mammalian lineage, while *opn6*, *7* and *8* are present in non-mammalian tetrapods and teleost fish, and *opn9* is conserved only in teleost (Davies et al., 2015; Beaudry et al., 2017). The pressure selection associated with the evolutionary conservation of neuropsins is unknown. Additionally, in the retina when neuropsin expression initiates developmentally the cell types that express the different neuropsins, and when these cells become integrated into light-activated circuits is poorly understood. This knowledge is important to our understanding of neuropsin physiology and the evolutionary processes which favoured particular neuropsins over others in the vertebrate retina.

The vertebrate retina detects light and converts it to neural signals that are sent to the brain to allow the organism to visualize its environment and regulate biological rhythms (Díaz et al., 2016; Thoreson and Dacey, 2019). Common to the retina of all vertebrates are five specialized types of neurons organized in three layers: (i) photoreceptors (rods and cones) located in the outer nuclear layer (ONL); (ii) horizontal (HC), bipolar (BC) and amacrine (AC) cells in the inner nuclear layer (INL); and (iii) retinal ganglion cells (RGCs) in the ganglion cell layer (GCL) (Lamb, 2013). Light entering the eye stimulates photoreceptors that then transmit electrical signals to RGCs through BCs. RGC axons exit the eye as the optic nerve and terminate at downstream brain targets. HC and AC interneurons modulate the neural signals within the retina. Muller glia (MG) are the predominant macroglial cells of the vertebrate neural retina and serve mainly in retinal support (Morera et al., 2016; Hoang et al., 2020).

Previous expression analyses localize neuropsins to particular retinal laminae (Davies et al., 2015), with only a few studies identifying the specific cell types that express the different neuropsins (Yamashita et al., 2010; Rios et al., 2019; D’Souza et al., 2022). In mouse, a *cre* recombinant system shows that *opn5* is expressed exclusively in RGCs (Calligaro et al., 2022; D’Souza et al., 2022), where it regulates retinal photoentrainment (Buhr et al., 2015). In birds, however, *opn5m* (“m” for mammalian-like) mRNA is additionally present in INL cells during development and after hatching (Rios et al., 2019; Calligaro et al., 2022). In fish and amphibians, *opn5* is expressed mainly in the inner portion of the INL, with a small subset of *opn5^+^* cells located in the GCL (Davies et al., 2015; Sato et al., 2016; Bertolesi et al., 2021). Less is known about the retinal expression of *opn6*, *7*, *8* and *9* that do not have mammalian counterparts. In *Xenopus laevis*, o*pn6*, a paralogue of *opn5*, underwent gene duplication to produce *opn6a* and *opn6b.* In the mature larval retina, we find both genes expressed in a handful of ONL cells that border the ciliary marginal zone (CMZ) (Bertolesi et al., 2021). These cells are likely newborn photoreceptors that arise from the CMZ, a proliferative neuroepithelium characteristic of fish and amphibians that provides new neurons for the retina over the life span of the organism (Perron and Harris, 1999). *opn6* mRNA is expressed by photoreceptors of the adult zebrafish retina (Davies et al., 2015). The amphibian *opn8* gene and its homologues in bony fish (*opn8a*, *opn8b* and *opn8c*) are detected in cells of the INL and GCL (Davies et al., 2015; Bertolesi et al., 2021), while *opn9* is expressed by cells of the outer INL of the mature teleost (Davies et al., 2015). These findings suggest that the spatial localization of neuropsin expression may differ between vertebrate lineages and species.

*X. laevis* is an excellent model organism to study how the function of neuropsins within the retina may compare to those of other opsins. First, the *neuropsins* and other *opsin* genes are identified (Chang and Harris, 1998; Bertolesi et al., 2020). After accounting for the duplication of genes resulting from the allotetraploid nature of *X. laevis*, there exists 22 distinct type-II *opsin* genes (Session et al., 2016; Bertolesi et al., 2020). Of the type-II opsins, the neuropsin family includes *opn5, opn6a, opn6b*, *opn7a*, *opn7b* and *opn8* (Bertolesi et al., 2020). The *opn7* genes, however, exhibit little or no expression in the retina at stage 43/44 (Bertolesi et al., 2021). Second, retinal neurogenesis and lamination, and the onset of expression of the classical opsins (rhodopsin and cone opsins) and melanopsins (*opn4* and *opn4a*; a.k.a. as *opn4m* and *opn4x* for mammalian- and *Xenopus*-like, respectively) are characterized in the developing *Xenopus* retina (Table 1). The expression of the classical opsins in the mature larval *Xenopus* retina (stage 43/44 tadpoles, mature, functional retina) and in adults is also known. For example, reports have defined both the distribution of the classical opsins in cones and rods in the ONL, and of *opn4*/*a* in HCs and RGCs (Table 1) (Chang and Harris, 1998; Gábriel and Wilhelm, 2001; Bertolesi et al., 2014).

**Table 1:** Timeline of eye development, onset of “classical” opsin and melanopsins and opsin expression/distribution in the *Xenopus laevis* “mature” retina.

Stage	Cell (*opsin*)	Proportion	Development
24			Retinal differentiation begins Holt et al. (1988)
31	BC		BC (serotoninergic) differentiation initiates Frederick et al. (1989)
33/35	HC/AC		HC (GABAergic) and AC (glycinergic and dopaminergic) differentiate Hollyfield et al. (1980)
33/34	PR		Lamination occurs and photoreceptors migrate to the ONL Holt et al. (1988)
33/34	PR (Rho/*sws1*)		Expression of classical opsins initiates Chang and Harris (1998)
32	HC/RGC (*opn4*/*opn4a*)		Melanopsin expression initiates Bertolesi et al. (2014)
35	PR		Photoreceptor differentiation well pronounced in dorsal retina Chang and Harris (1998)
35/36	MG		Initial detection of MG cells (GFAP^+^ immunoreactivity) Szaro and Gainer (1988)
37/38	AC/BC/RGC		First retinal circuits turn on (light-induced *c-fos* expression) Bertolesi et al. (2014)
39/40	RGC		RGC axons target the optic tectum Holt (1989)
41			Neurogenesis stops except by mitotic cells of the ciliary marginal zone and MG Holt et al. (1988)

Stage	Cell (*opsin*)	Proportion	Mature retina
43/44			Fully functional retina
43/49			Retinotectal circuits continue developing Liu et al. (2016)
43-A	PR		Similar proportion of rods and cones in the ONL Gábriel and Wilhelm (2001) and Bertolesi et al. (2021)
43-A	PR (*rho*)	~47%–53%	Rhodopsin expression in red rods Chang and Harris (1998) and Bertolesi et al. (2014)
43-A	PR (*sws1*)	~2%–3%	Green rods from amphibians (e.g., anurans) express the cone opsin *sws1* Takahashi et al. (2001), Kojima et al. (2017), Bertolesi et al. (2021) and Gábriel and Wilhelm (2001)
43-A	PR (*lws1*)	~40%	Cones expressing *lws1* Bertolesi et al. (2021) and Gábriel and Wilhelm (2001)
43-A	PR (*sws2*)	~10%	Cones expressing the blue-light-sensitive opsin *sws2* Gábriel and Wilhelm (2001) and Bertolesi et al. (2021)
43	PR (*sws2*/*opnp*)	~1	Small proportion of cones (*sws1^+^*) also express *pinopsin* (*opnp*) Bertolesi et al. (2021)
43	HC/RGC (*opn4*/*a*)		~7:1 ratio of HC:RGC co-express *opn4* and o*pn4a* Bertolesi et al. (2014, 2021)

A, adult or juvenile.

Here, we determined when *neuropsin* expression begins in the embryonic *X. laevis* retina and identified the cell types involved. Additionally, we investigated co-expression between *neuropsins* and *opn4*, and the activation of neuropsin-expressing cells in light-stimulated circuitry by looking at expression of the immediate early gene marker *c-fos* (Yu et al., 1994; Huerta et al., 1997). Finally, we compared our findings in *X. laevis* to other species with publicly available whole-eye single-cell RNA-sequencing (scRNA-seq) data. For *Xenopus*, this powerful technique was used to study gene expression and cell type diversity during embryogenesis (Briggs et al., 2018; Sonam et al., 2022), though retinal data is not yet publicly available. Gene expression, however, was recently analyzed for adult zebrafish, chicken and mouse retinas (Hoang et al., 2020). We compared our data from larval *X. laevis* with that of a scRNA-seq dataset from the adult zebrafish retina (Hoang et al., 2020) to assess differences in neuropsin expression between vertebrate lineages. Our results contribute to the understanding of retinal neuropsins in two novel aspects: (1) by comparing with the known expression of opsins in the *Xenopus* retina, we provide insight into neuropsin physiology. Specifically, we describe new photoreceptor cell types and neuropsin-expressing cells in the INL and GCL, the developmental time-course of expression, and whether expressing cells are integrated into light-activated circuits; (2) by comparing *neuropsin* expression between different species we provide insight into evolutionary differences between *neuropsins*.

## Materials and methods

### Embryos

All procedures involving frogs and tadpole embryos were approved by the Animal Care and Use Committee at the University of Calgary. *X. laevis* egg production was induced by chorionic gonadotrophin injection (Intervet Canada Ltd.) and *in vitro* fertilization in accordance with standard procedures (see protocols at www.xenbase.org/ RRID:SCR_003280). Embryos were stored at 16°C or 22°C and were kept on a 12:12 h cycle of light and dark. For light exposure experiments, embryos were kept in the dark from stage 24 until they reached stage 43 and were exposed to 30 min of white light (approximately 800 lux) immediately before fixing. Embryo stages were determined using Nieuwkoop and Faber’s developmental data for *X. laevis* stages on Xenbase.^1^

### Single RNA *in-situ* hybridization

Embryos at desired stages were collected and fixed in MEMFA [(0.1 M 3-N-morpholino propanesulfonic acid (MOPS), 2 mM ethylene glycol tetra-acetic acid (EGTA), 1 mM MgSO_4_, and 4% formaldehyde in diethylpyrocarbonate (DEPC)-treated H_2_O)] at 4°C. After 24 h, embryos were placed in 30% sucrose in DEPC water until sunken and then frozen in optimal cutting temperature (OCT) compound (Tissue Tek, Sakura Finetek Inc., United States) and stored at −80°C. OCT-embedded embryos underwent cryostat sectioning (12 μm thick sections) for slide *in situ* hybridization (ISH). Sense and antisense riboprobes were synthesized from linearized plasmids containing the partial sequences of *X. laevis opn4a, opn5*, *opn6a*, *opn6b*, *opn8*, and *c-fos* (GenBank access numbers: MN820837, MN820850, MN820855, MN820856, MN820855 and BC079689.1, respectively). SP6 and T7 RNA polymerases (Roche, Quebec, Canada) with digoxigenin (DIG)-labeled nucleotides (Roche) were used to generate the riboprobes. Riboprobes were hybridized at 65°C and detected with anti-DIG antibodies (1:2500, Roche) conjugated to alkaline phosphatase, and the signal developed with BCIP (Roche; 5-bromo-4-chloro-3-indolyl phosphate) and NBT (nitro blue tetrazolium chloride; Roche) in a NTMT solution (100 mM NaCl, 100 mM Tris-Cl pH 9.5, 50 mM MgCl_2_, and 1% Tween 20).

### Double fluorescent *in-situ* hybridization (FISH)

DIG- and fluorescein isothiocyanate (FITC)-labeled probes were synthesized and detected by using anti-DIG (1:500 dilution) and anti-FITC (1:500 dilution) specific antibodies coupled to horse radish peroxidase. The signal was developed with tyramide signal amplification (TSA) cyanine 3 or TSA fluorescein system kits, respectively (both Perkin Elmer, United States), following the manufacturer’s protocol.

### Immunohistochemistry post fluorescent *in-situ* hybridization

DIG-labeled probes developed with TSA cyanine 3 were used for single FISH of retina sections. Following single FISH, immunohistochemistry was performed by incubating the slides with antibodies for Otx2 (rabbit polyclonal 1:200; ab21990, Abcam), Pax6 (rabbit polyclonal 1:100, Cedarlane), Rhodopsin (clone 4D2; mouse 1:200; Millipore) or Calbindin (rabbit polyclonal 1:200; Swant) for 1 hour. Alexa Fluor-tagged secondary antibodies (1:1000, green, 488 nm excitation, anti-mouse or anti-rabbit, Molecular Probes) were used to identify the primary antibody. DAPI staining after ISH and immunohistochemistry labeled cell nuclei and guided cell counting.

### Microscopy

For single RNA ISH, bright field optics on a Zeiss compound microscope were used to examine slides through a 20× or 40× objective. Digital images were obtained with Axiovision software. A Zeiss LSM 900 confocal laser scanning microscope was used to image both FISH followed by immunohistochemistry, and double FISH. Z-stacks of digital images (1 μm thick) were generated by using Zen Blue software (Zeiss) on the confocal microscope. A minimum of 8 embryos (*N*) were analyzed for single ISH, FISH, double FISH, or FISH followed by immunohistochemistry.

### Single cell RNA sequencing analysis

Publicly available single cell RNA sequencing (scRNA-seq) whole eye datasets for the adult zebrafish (*Danio rerio*), chicken (P10) (*Gallus gallus*) and mouse (P60) (*Mus musculus*) were downloaded from GitHub at https://github.com/jiewwwang/Single-cell-retinal-regeneration (Hoang et al., 2020). For each species, RStudio (R version 4.3.0) and Seurat V3 were used to generate a Seurat object. All metadata, including cell annotations and sample identification, were preserved from the original study. The Seurat objects were subset to include only control (uninjured) retinae. Quality control was performed to include only cells with between 500 and 2,500 gene features (standard values) and less than 15% mitochondrial genes. A standard Seurat integration pipeline with the “vst” selection method and 2000 variable features was performed for each species. Integrated data were scaled, and an unbiased principal component analysis was performed. RunUMAP was performed with 19 dimensions determined by JackStraw statistics. Original annotation was re-evaluated and confirmed by examining the expression of common cell type markers (Supplementary Table S1) in UMAP clusters indicated by the “FindAllMarkers” function in Seurat.

Neuropsin expression in distinct clusters was evaluated using a custom R script which parses through a gene count matrix to find the number of cells of a particular cell type that express a gene at or above an expression level of one UMI. A list of genes was provided to the script which were then iteratively analyzed, and a data-frame was generated for all results. Github repository including all information pertaining to the downloading and instructions for how to use the function of the “blackshaw” dataset is linked to the following website https://github.com/s-storey/opn_expression/tree/main.

### Cell counting and analysis

Digital images from confocal microscopy that captured expression in single transverse central retinal sections containing the optic nerve were collected for analysis of neuropsin-positive cells in the retina. If the optic nerve section was not preserved, an adjacent anterior or posterior section was used to count cells The number of retinal slices analyzed (*n*) and the mean with 95% confidence interval are indicated in the corresponding figures. Immunohistochemical analysis were performed at less two times (*N* ≥ 2) containing a minimum of 4 independent tadpoles in each experiment. The number of central retina sections analyzed (*n*) is indicated in figure legends. GraphPad Prism software (v.9.1.2, GraphPad Software, San Diego, CA, United States) was used for statistical analysis.

## Results

### Developmental expression of neuropsins in the *Xenopus* retina

**Figure 1:**
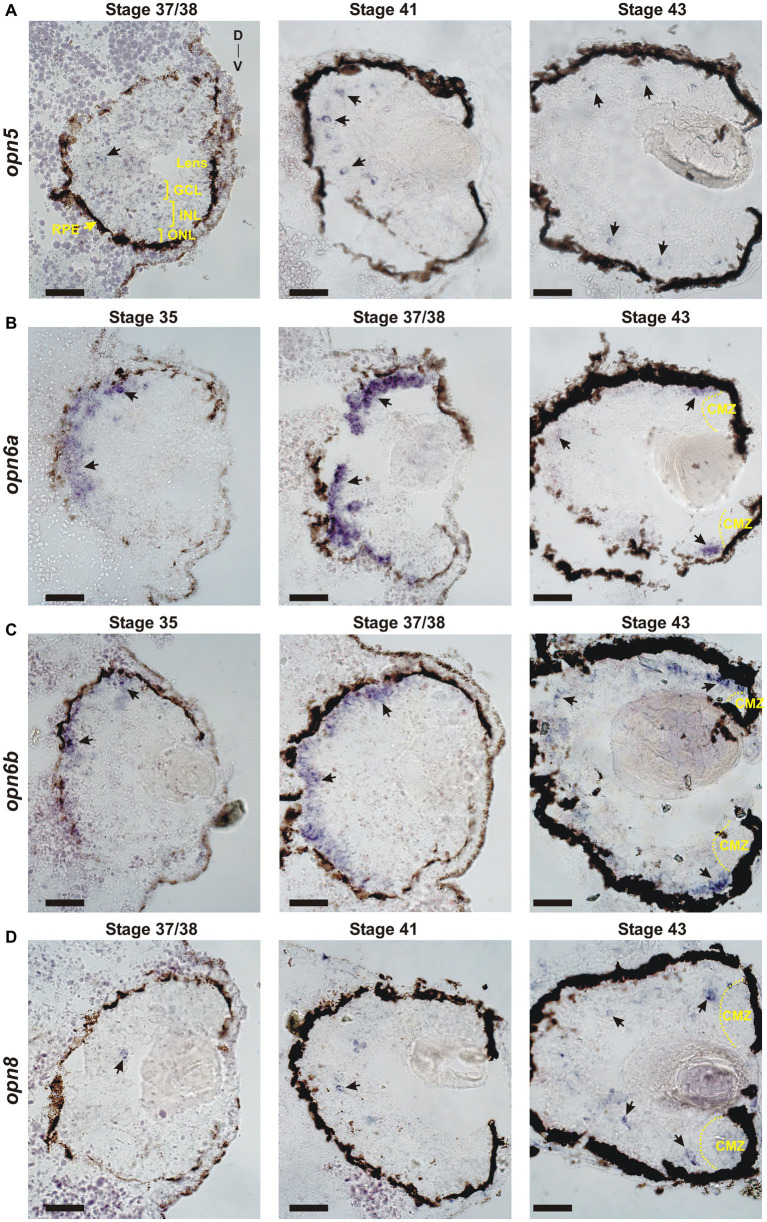
Developmental expression of *neuropsins*. ISH with specific antisense riboprobes against *opn5* **(A)**, *opn6a* **(B)**, *opn6b* **(C)** and *opn8* **(D)** on transverse retinal sections at stages 35, 37/38, 41 and 43. Black arrows point to neuropsin-expressing cells. CMZ, ciliary marginal zone; GCL, ganglion cell layer; INL, inner nuclear layer; ONL, outer nuclear layer; RPE, retinal pigment epithelium; D, dorsal; V, ventral. Scale bar = 100 μm.

**Figure 2:**
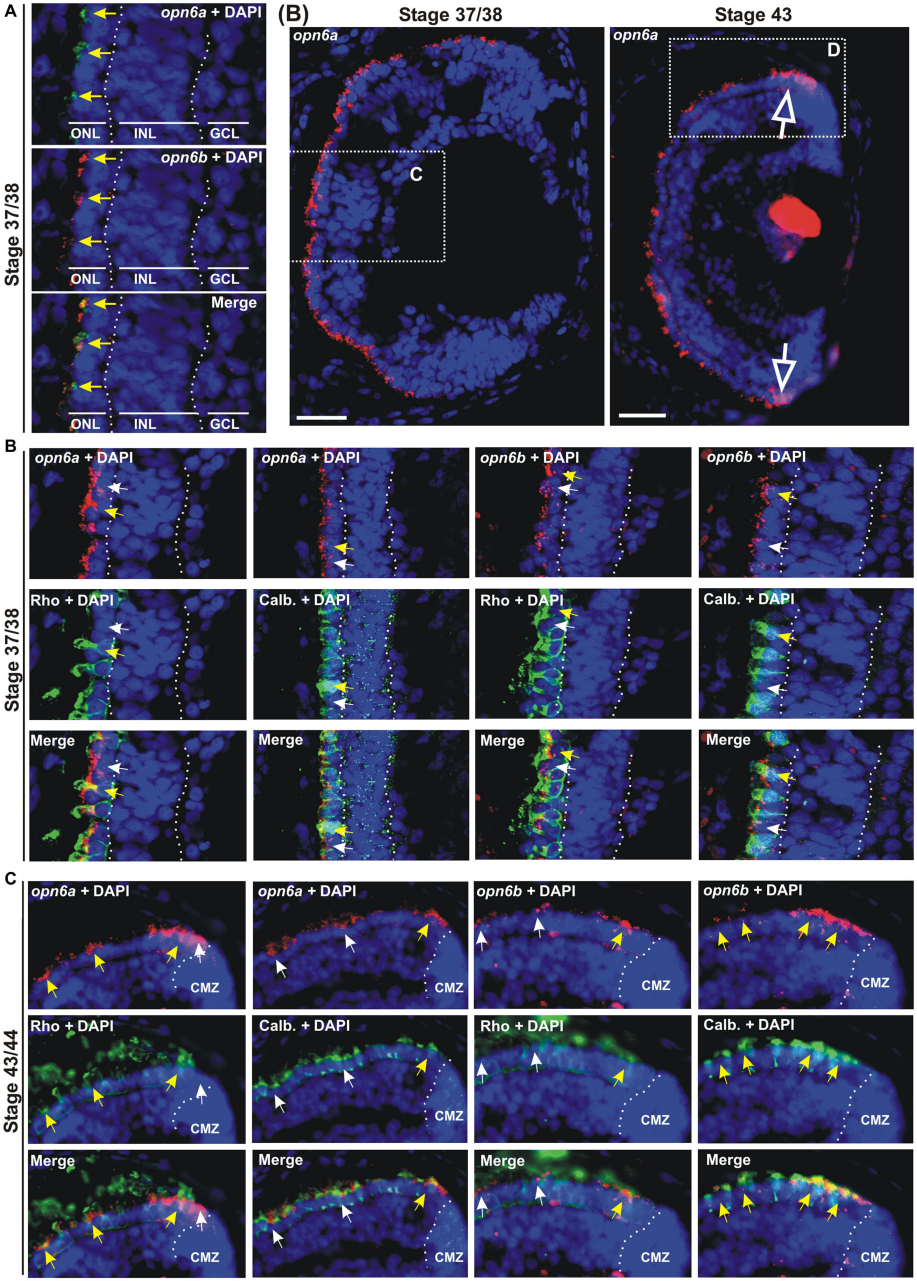
*opn6a* and *opn6b* colocalize in newly born photoreceptors at stage 37/38 but are differentially expressed in differentiated rods and cones at stage 43/44. **(A–D)** Fluorescent ISH with specific riboprobes against *opn6a* or *opn6b* on stage 37/38 **(A,C)** and 43/44 *Xenopus laevis* transverse retinal sections **(B,D)**, followed by immunohistochemistry against a rod marker (rhodopsin; green) or a cone marker (calbindin; green) **(C,D)**. DAPI (blue) stains nuclei. **(A)** Yellow arrows point to ONL cells that express both *opn6a* and *opn6b*. **(B)** Higher magnification of the dotted-line boxes are shown in bottom panels **(C,D)**. The open white arrows point to *opn6a^+^* cells that are adjacent to the ciliary marginal zone (CMZ). **(C,D)** White small arrows point to *opn6a* or *opn6b* expressing cells that are either not rhodopsin^+^ or not calbindin^+^, while yellow arrows indicate *opn6a*^+^ or *opn6b*^+^ cells that are also positive for rhodopsin or calbindin. GLC, ganglion cell layer; INL, inner nuclear layer; ONL, outer nuclear layer. Scale bar in **(B)**: 100 μm.

The onset of *neuropsin* expression in the retina is unknown. We analyzed the developmental expression of retinal neuropsins found in *X. laevis*: *opn5, opn6a, opn6b* and *opn8* (Bertolesi et al., 2020, 2021). Neuronal differentiation initiates at stage 24 (1 day of development), classical opsins are detected a day later at stage 35, and the RGC axons target the optic tectum by stage 39/40 (3 days of development) (Table 1). Two *opn5* genes exist in *X. laevis.* These genes likely originated from the *X. laevis* specific tetraploid in that both localize to homologous chromosome 5; *opn5.L* (gene number 100500792) and *opn5.S* (gene number 100500792) located on homologous chromosomes “long” and “short,” respectively, which correspond to the sub-genomes (2*n* each) provided by the distinct progenitors that gave rise to the tetraploid. ISH on 12 μm retinal sections using an *opn5.L* riboprobe (1,222 bp length; GenBank number: MN820850) revealed a specific, yet sparse, expression pattern first detected at stage 37/38 (Figure 1A), when the retinal circuits initially become light-activated (Bertolesi et al., 2014). At stage 41, when the retinal-brain circuits are functional, *opn5* expression was strongest in cells located in the outer region of the INL (Figure 1A). By stage 43, *opn5* was expressed sparsely in the INL, and several cells (~20% of total *opn5^+^* cells) were present in the GCL (Figure 2). Of note, the *opn5.L* probe possessed 91% identity to *opn5.S* (GenBank number XM_018264817.1), therefore expression of both *opn5* genes was likely detected. *opn5* expression in the INL and GCL in *X. laevis* resembles the pattern reported previously in post-hatching chick retina (Yamashita et al., 2010), but differs from mammals, where *opn5* expression appears restricted to the GCL (Calligaro et al., 2022; D’Souza et al., 2022).

We previously identified three *opn6* genes in *X. laevis* that are phylogenetically related, and that we named *opn6a.L*, *opn6a.S*, and *opn6b.L* (Bertolesi et al., 2020). Two homologous genes exist for *opn6a,* located on the homologous chromosomes 5 L and 5 S (*opn6a.L*, gene number 108716317 and *opn6a.S*, gene number 108717820), while *opn6b.L* (gene number 108698982) is located on chromosome 8 L as a single copy. *opn6a* and *opn6b* are not homologous genes resulting from *X. laevis* tetraploidy. Instead *opn6b.L* was potentially generated by inter-chromosomal transposition. The probe generated to detect *opn6a* (972 bp) was produced from the *opn6a.L* template (GenBank number MN820855) and contained 94% identity with the predicted *opn6a.S* transcript sequence. Therefore, the *opn6a* probe likely recognized both *opn6a.L* and *opn6a.S* homologous transcripts. In contrast, no significant similarity was found between the recognition sequence of the *opn6a.L* probe and that of *opn6b.L* (gene number 108698982). Furthermore, the sequence recognized by the *opn6b* probe (1,047 bp length) showed no significant similarity to *opn6a.L* or *opn6a.S*. Of note, at the time of this publication, *opn6b.L* is classified as an *opn5* gene in the NCBI database and should be renamed.

*opn6a* expression was first detected at stage 35 as diffuse staining, mainly in cells of the dorsal-most outer layer of the retina, with a few positive cells located in the inner portion of the retina (Figure 1B). By stage 37/38, *opn6a* was expressed strongly by cells of the ONL (Figure 1B). *opn6a* was present in the ONL by stage 43, but apparently in a smaller number of cells than at stage 37/38, with the strongest expression observed in cells that border the CMZ (Figures 1B, 2B). The CMZ-associated expression at stage 43/44 is consistent with our previous data suggesting that *opn6a* is robustly expressed in a transient manner in newborn photoreceptors (Bertolesi et al., 2021). Interestingly, the *opn6b* expression was similar to *opn6a*, re-enforcing the idea that *opn6b* is related to *opn6a* and not to *opn5*. *opn6b* expression was first detected at stage 35, and most strongly expressed at stage 37/38 in the ONL. Fewer cells expressed *opn6b* mRNA in the ONL by stage 43, with the strongest label located at the CMZ border (Figure 1C). Interestingly, the developmental time-course of *opn6a*/*b* expression at stage 35/36 and 37/38 matches the characterized processes of specification, migration, and differentiation of *X. laevis* cone and rod photoreceptors (Table 1) (Chang and Harris, 1998). The decrease in expression seen in maturing photoreceptors integrated into functional retinal circuits (stages 41 and 43/44), however, is different from what is observed for the classical opsins that continue to be highly expressed with ongoing larval development (Chang and Harris, 1998).

We detected three related *opn7* genes in *Xenopus laevis*: *opn7a.L* and *opn7b.L* and *opn7b.S* (gene numbers 108718575, 108700890 and 108702595, respectively). *opn7b.L* and *opn7b.S* are homologous genes located on chromosomes 9–10 L and S, respectively, while *opn7a.L* is located on chromosome 6 L (Bertolesi et al., 2020). mRNA for these genes is almost undetectable in the eye at stage 43/44 (Bertolesi et al., 2021), and therefore we did not analyze them here.

Two *opn8* genes (*opn8.L*; gene number 108717224 and *opn8.S*; gene number 108718281) are located on chromosome 5 L and 5 S, respectively. Expression of the *opn8* genes was analyzed by ISH using a probe generated against *opn8.L* (904 bp length; GenBank number MN820854; with 91% identity to *opn8.S*). *opn8* expression was first detected at stage 37/38 in a few cells located in the INL. *opn8* remained sparsely expressed in the INL, GCL and in cells bordering the CMZ with ongoing development (stage 41 and 43) (Figure 1D).

Together, our results show a specific pattern of neuropsin expression in the retina. *opn6a* and *opn6b* are expressed by photoreceptors in the ONL, with expression beginning about the same time as the classical opsins at ~stage 35. With further development, however, *opn6a* and *opn6b* expression diminishes in fully differentiated photoreceptors, but is present likely in newborn photoreceptors that arise from the CMZ. The expression of *opn5* and *opn8* turns on slightly later at stage 37/38. In the mature larval retina (stage 43), *opn5* and *opn8* are both expressed mainly in cells located in the INL, with the remainder of cells in the GCL.

### *opn6a* and *opn6b* mRNAs initially colocalize in rods and cones, but in the maturing larval retina become differentially expressed by rods and cones, respectively

Next, we identified the specific cell types that express the different neuropsins. We started with the analysis of which photoreceptors in the ONL express *opn6*. Our ISH data show strong *opn6a* and *opn6b* expression at stage 37/38. Thus, we chose this stage to determine whether these two opsins are co-expressed and in which photoreceptor classes. Co-expression was determined by double fluorescent ISH (dFISH). At stage 37/38, most photoreceptors appeared to express both *opn6a* and *opn6b* (Figure 2A). Cell types were assigned through FISH, followed by immunohistochemistry either against the known rod marker, rhodopsin, or calbindin, a calcium binding protein expressed by cones (Chang and Harris, 1998). Initially, *opn6a* and *opn6b* were expressed by both rhodopsin^+^ and calbindin^+^ cells, with the fluorescent signal commonly localizing to the inner segments of photoreceptors as a cluster of fluorescent dots (Figures 2B,C). However, by stage 43/44, once retinal circuits are functional and connected to the brain, *opn6a* mRNA was found preferentially in differentiated rods (rhodopsin^+^), and in only a few differentiated cones (calbindin^+^) (Figures 2B,D). In the opposite manner, *opn6b* mRNA was found preferentially in differentiated calbindin^+^ cones and only a few differentiated rhodopsin^+^ rods (Figure 2D). Interestingly, for the newly born photoreceptors located proximal to the CMZ *opn6* opsins were expressed in both rhodopsin^+^ and calbindin^+^ cells, similar to what was observed in the central retina at a younger developmental stage (stage 37/38) (Figure 2D).

We find *opn6a* and *opn6b* are co-expressed by all newly generated photoreceptors shortly after their birth, either in the central embryonic retina (stage 37/38) or those generated subsequently by the CMZ (stage 43). With differentiation in the central retina, however, *opn6a* and *opn6b* become selectively expressed in rod and cone photoreceptors, respectively.

### *opn5* is expressed by bipolar and amacrine cells, *opn8* is expressed solely by amacrine cells, and Pax6^+^ RGCs express both opsins

Next, we identified the cell types that express *opn5* and *opn8* in the INL and GCL. The ISH data indicated that by stage 41, when retinal differentiation and circuity are defined (Table 1), *opn5* and *opn8* are mainly expressed in the INL and a subset of cells in the GCL. To determine the cell types located in the INL, we performed FISH followed by immunohistochemistry against the transcription factors Otx2 and Pax6 that in the INL label BCs (Viczian et al., 2003) and ACs (Hirsch and Harris, 1997), respectively.

**Figure 3:**
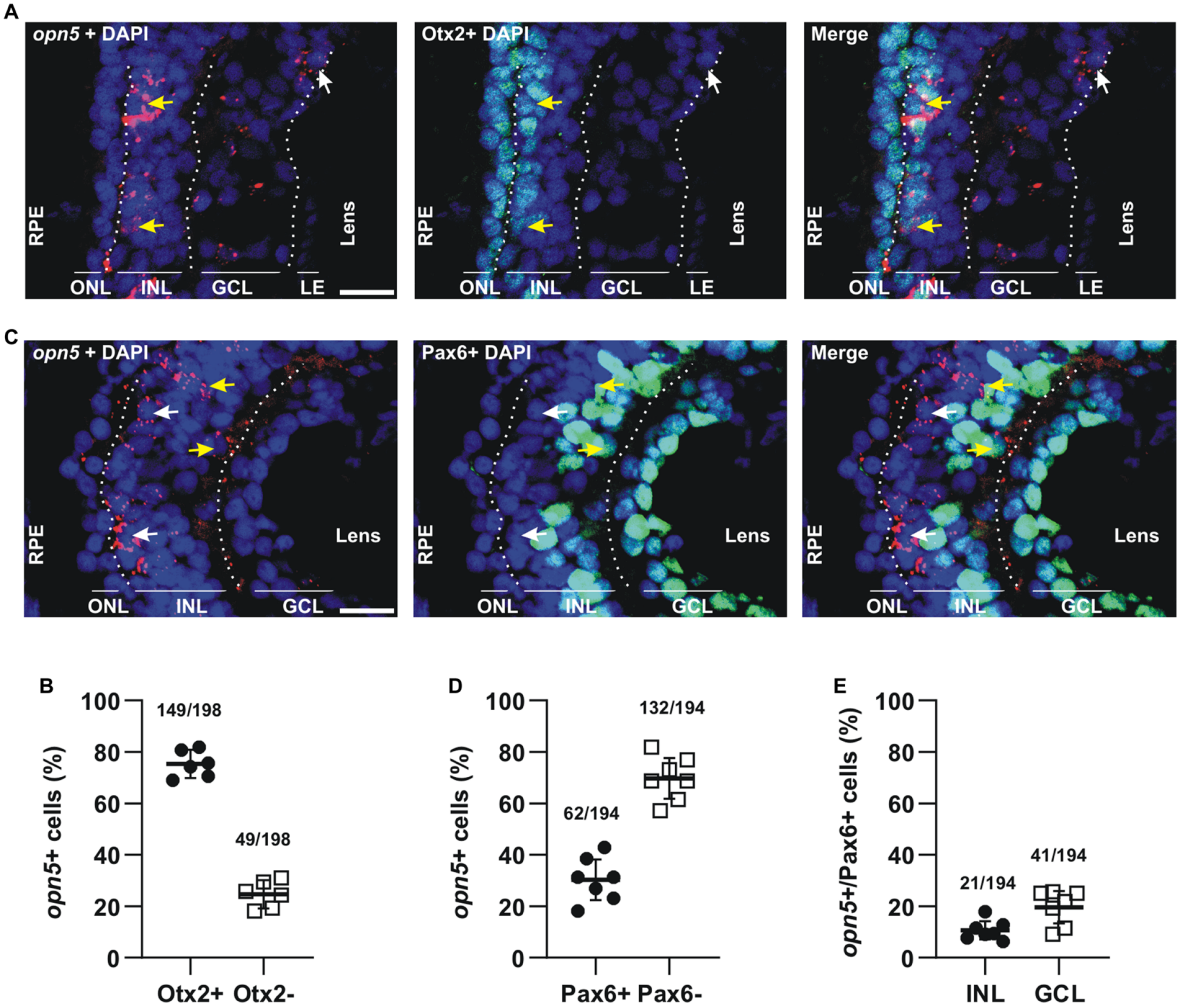
*opn5* is expressed in bipolar and amacrine cells in the INL and in RGCs. **(A,C)** FISH against *opn5* (red) followed by immunohistochemistry against a bipolar cell marker (Otx2; green) **(A)** or an amacrine and retinal ganglion cell marker (Pax6; green) **(C)**, on stage 41 *Xenopus laevis* transverse retinal sections. DAPI (blue) stains the nucleus. ONL (outer nuclear layer), INL (inner nuclear layer), and GCL (ganglion cell layer) are indicated between dotted lines. White arrows point to *opn5*-expressing cells that are either Otx2^−^ or Pax6^−^, while yellow arrows indicate *opn5*^+^ cells that are also Otx2^+^ or Pax6^+^. RPE, retinal pigment epithelium. Scale bar = 50 μm. **(B,D,E)** Graphs showing percentage of *opn5*^+^ cells that are Otx2^+^ **(B)** or Pax6^+^ **(D)**, and the distribution of Pax6^+^/*opn5*^+^cells between the INL and GCL of stage 41 retina **(E)**. Each dot represents the percentage of cells in one central retina section [**(B)**, *n* = 6 sections; **(D,E)**, *n* = 7 sections]. Lines are the mean with 95% confidence interval.

*opn5* mRNA was seen by FISH as clusters of subcellular red fluorescent dots that were either nuclear or associated with the nuclear membrane (Figure 3A). Most of the *opn5^+^*cells were in the outer portion of the INL (Figures 1, 3A). Approximately 70%–75% of the *opn5^+^* cells were BCs, as they colocalized with Otx2 (75.25%, 149 *opn5*^+^ Otx2^+^ cells/198 *opn5^+^* cells; *n* = 6 retinal sections) (Figures 3A,B). The remaining populations (24.74%, 49 *opn5*^+^ Otx2^−^ cells/198 *opn5^+^* cells) were distributed either in the inner part of the INL or the GCL (Figures 3A,B). We found that these cells were Pax6^+^ (Figure 3C); the *opn5*^+^ Pax6^+^ cells in the INL were ACs (10.8%, 21 cells *opn5^+^* Pax6^+^ in the INL/194 *opn5^+^*cells; Figure 3D), and the remaining were located in the GCL (21.13%, 41 cells *opn5^+^* Pax6^+^ in the GCL/194 *opn5^+^*cells, Figure 3E).

**Figure 4:**
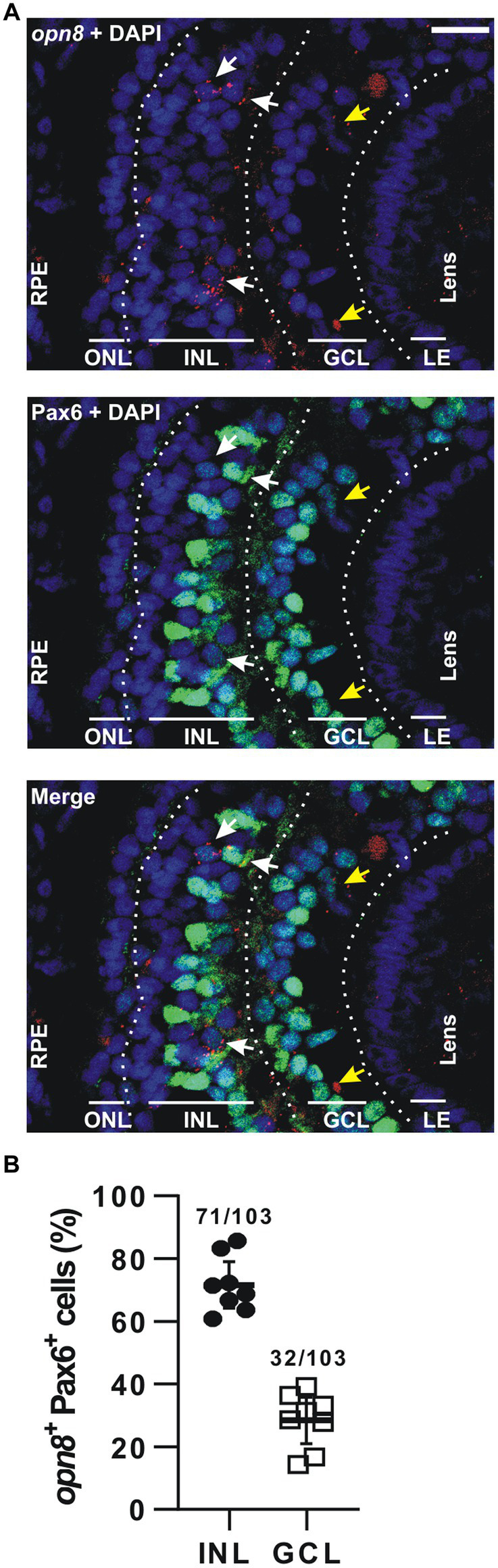
*opn8* is expressed in Pax6^+^ cells in the INL and GCL. **(A)** Fluorescent ISH against *opn8* (red) followed by immunohistochemistry against an amacrine and RGC marker (Pax6; green) on stage 41 *Xenopus laevis* transverse retinal section. DAPI (blue) stained the nucleus. ONL, INL, and GCL indicated with bars. White arrows point to *opn8*-expressing cells that are Pax6^+^ INL cells, while yellow arrows indicate Pax6^+^ GCL cells. GCL, ganglion cell layer; INL, inner nuclear layer; ONL, outer nuclear layer; RPE, retinal pigment epithelium. Scale bar = 50 μm. **(B)** Distribution of *opn8^+^*/Pax6^+^ cells between the INL and the GCL of stage 41 retinas. Each dot represents the percentage of cells in one central retina section (*n* = 8 sections); Lines are the mean with 95% confidence interval.

For *opn8-*expressing cells, we used immunohistochemistry against Pax6 after FISH at stage 41, when *opn8* is expressed in both the INL and the GCL. Like *opn5*, *opn8* expression was seen by FISH as subcellular red fluorescent dots that were either nuclear localized or associated with a nuclear membrane (Figure 4A). *opn8* was expressed by only a few retinal cells per section. These cells were all Pax6^+^ and were found in either the AC-populated inner portion of the INL or the GCL (Figures 4B,C). Thus, for the *opn8^+^* cells, 70% were INL-located cells, likely ACs, and 30% were GCL-located cells (Figure 4B).

### Neuropsins and melanopsin show distinct and partially overlapping expression in retinal cells

**Figure 5:**
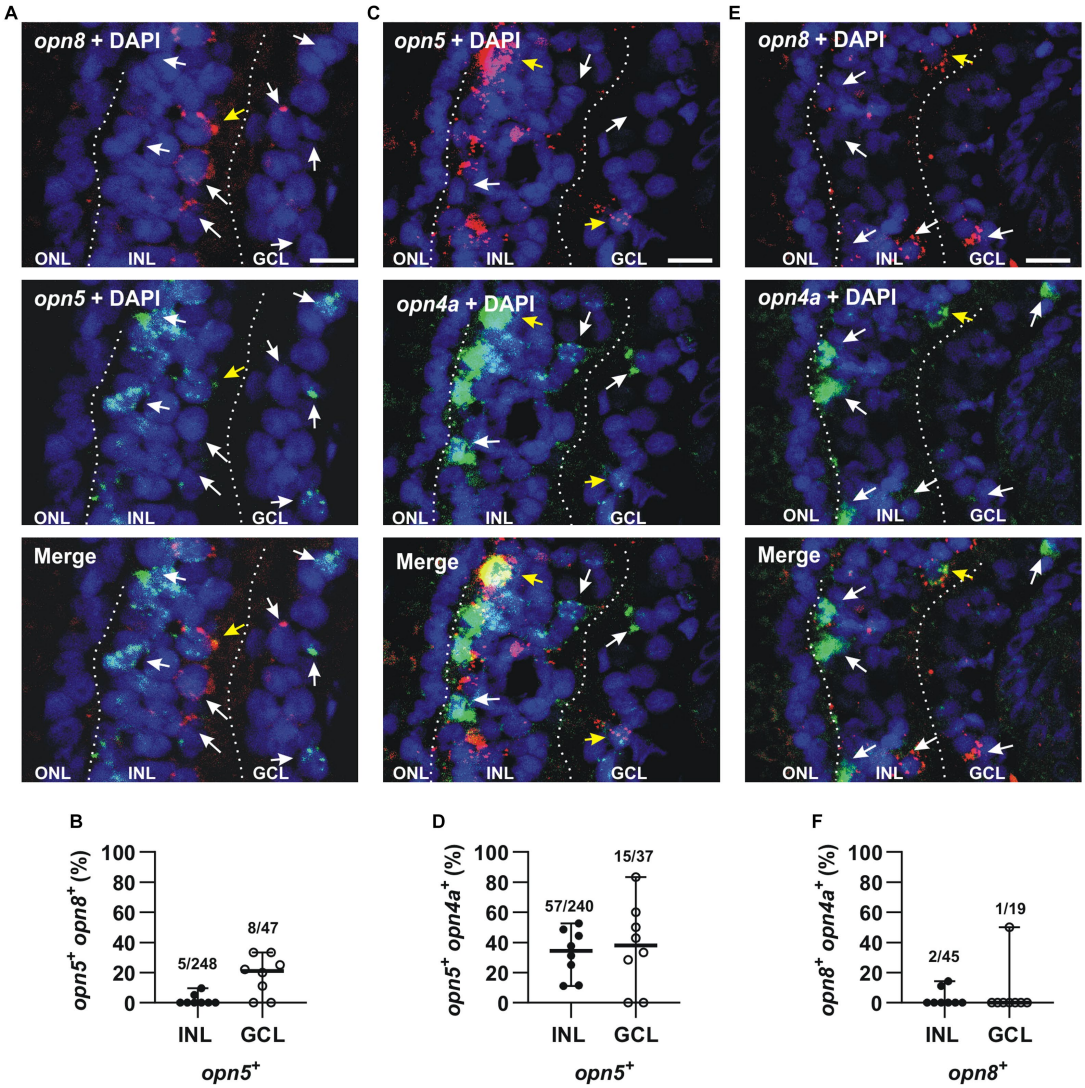
*opn5* and *opn8* sparsely co-express in the GCL but not INL, while *opn4* and *opn5* are partially co-expressed in both GCL and INL, and *opn4* and *opn8* do not co-express. **(A,C,E)** Double FISH against *opn5* (green) and *opn8* (red) **(A)**, *opn5* (red) and *opn4a* (green) **(C)**, and *opn8* (red) and *opn4a* (green) **(E)** on stage 43/44 *X. laevis* transverse retinal sections. DAPI (blue) stained the nucleus and is shown with the merged image. White arrows point to cells that express a single opsin, while yellow arrows point to cells that express two opsins. GCL, ganglion cell layer; INL, inner nuclear layer; ONL, outer nuclear layer. Scale bar = 20 μm. **(B,D,F)** Graphs showing distribution between the INL and GCL of cells that co-express *opn5* and *opn8* **(B)**, *opn5* and *opn4a* **(D)** and *opn8* and *opn4a* **(F)**. Each dot represents the percentage of cells in one central retina section [*n* = 8 sections, **(B,D,F)**]. Lines are the mean with 95% confidence interval.

The expression of *opn5* and *opn8* in Pax6^+^cells in the INL and the GCL led us investigate possible neuropsin co-expression by performing double ISH against *opn5* and *opn8* on stage 43/44 retinal sections. Interestingly, we found that *opn5* and *opn8* mRNAs did not colocalize in the INL (2% co-localization; 5 cells *opn5^+^ opn8^+^*/248 *opn5^+^* cells; 52 *opn8^+^* cells counted) and only a few cells expressed both genes in the GCL (17% co-localization; 8 cells *opn5^+^ opn8^+^*/47 *opn5*^+^ cells; *n* = 8 retinal sections) (Figures 5A,B).

We also analyzed if neuropsins were co-expressed with *melanopsin*. In the *Xenopus* retina, *opn4* and *opn4a* are co-expressed (91% colocalization) in a subset of HCs and RGCs (Bertolesi et al., 2014). Because of the high level of co-expression, we could use the *opn4a* antisense probe alone in order to determine both *opn4* and *opn4a* colocalization with *opn5* and *opn8*. Double FISH at stage 43/44 revealed that only 24% of *opn5*^+^ cells also expressed *opn4a* in the INL (57 *opn5^+^ opn4a^+^*cells/240 *opn5^+^* cells), while 41% of cells expressed *opn4a* and *opn5* in the GCL (15 *opn5^+^ opn4a^+^* cells/37 *opn5^+^*cells; *n* = 8 retina central sections) (Figures 5C,D). In contrast, *opn8* was not expressed by *melanopsin*^+^ cells of the INL or GCL (4% in the INL; 2 cells *opn8^+^ opn4a^+^*/45 *opn8^+^* cells; *n* = 148 *opn4a^+^*cells counted; *n* = 8 retinal sections) (5% in the GCL; 1 cell *opn8^+^ opn4a^+^*/19 *opn8^+^* cells; *n* = 19 *opn4a^+^* cells counted in eight retinal sections) (Figures 5E,F).

Together, these results suggest that neuropsins are differentially expressed in distinct cells, with *opn5* in BCs, ACs, and RGCs, and *opn8* in ACs and in a different subset of RGCs. Additionally, mRGCs do not express *opn8* and only a few express *opn5*. Thus, our results suggest that the neuropsins *opn5* and *opn8* allow for a greater total number of potentially photosensitive RGCs.

### Light induces *c-fos* expression in *opn5*- and *opn8*-expressing cells

Next, we investigated whether the neuropsin-expressing cells participate in light-stimulated retinal circuits. To identify light-activated cells, we used *c-fos*, an immediate early gene marker expressed in neurons activated in response to synaptic stimuli (Yoshida et al., 1996; Huerta et al., 1997). We showed previously that several cells express *c-fos* after 30 min of white light illumination, particularly in the retina of dark-adapted *X. laevis* embryos (Bertolesi et al., 2014). Specifically, light-induced activation occurs in at least two cells of the INL (ACs and BCs) for each activated RGC. No *c-fos* expression is induced in photoreceptors (Bertolesi et al., 2014). Thus, we used double FISH to analyze at stage 43/44 the co-expression of *c-fos* with *opn5* and *opn8* upon light exposure.

**Figure 6:**
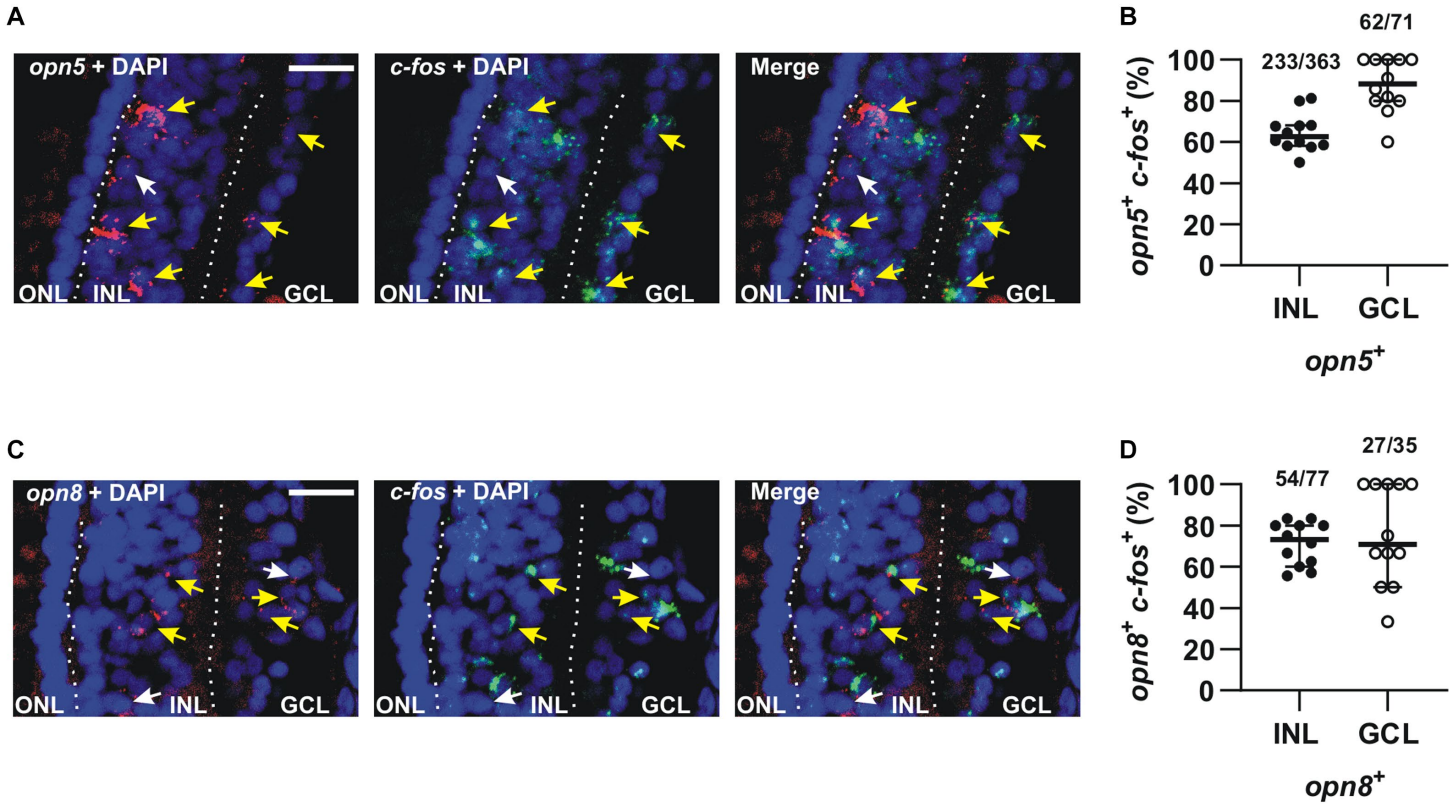
The majority of *opn5*^+^ and *opn8*^+^ cells were activated in response to light. **(A,C)** Double FISH against *opn5* (red) and *c-fos* (green) **(A)** and *opn8* (red) and *c-fos* (green) **(C)** on stage 43/44 *Xenopus laevis* transverse retinal sections after 30 min of light stimulation. DAPI (blue) stains the nucleus. White arrows point to *opn5*^+^ or *opn8*^+^ cells that do not express *c-fos*, while yellow arrows point to *opn5*^+^ or *opn8*^+^ cells that are also *c-fos*^+^. GCL, ganglion cell layer; INL, inner nuclear layer; ONL, outer nuclear layer. Scale bar = 50 μm. **(B,D)** Quantification and distribution between the INL and GCL of cells co-expressing *opn5* and *c-fos* (B) and *opn8* and *c-fos* **(D)**. Each dot represents the percentage of cells in one central retina section *n* = 12 sections; Lines are the mean with 95% confidence interval.

We found that the majority of *opn5*-expressing cells in the INL (64%) expressed *c-fos* (*n* = 363 cells counted) after 30 min of light stimulation, as did almost all cells (87%) in the GCL (*n* = 71 cells counted) (Figures 6A,B). Similarly, 70% and 77% of *opn8*-expressing cells also expressed *c-fos* in the INL (*n* = 77 cells counted) and the GCL (*n* = 35 cells counted), respectively (Figures 6C,D). It is worth noting that without light stimulation, almost no *c-fos* expression was detected in the retina of dark-adapted embryos (Supplementary Figure S1 and Bertolesi et al., 2014).

Together, our data show that the majority of *opn5*^+^ and *opn8^+^* cells in the INL and GCL express *c-fos* after light stimulation. We also define a second group of opsin-expressing RGCs, in addition to the larger number of melanopsin-expressing cells in the GCL (Bertolesi et al., 2014), which are in light-activated circuits.

### Expression of neuropsins in the retina of adult zebrafish

**Figure 7:**
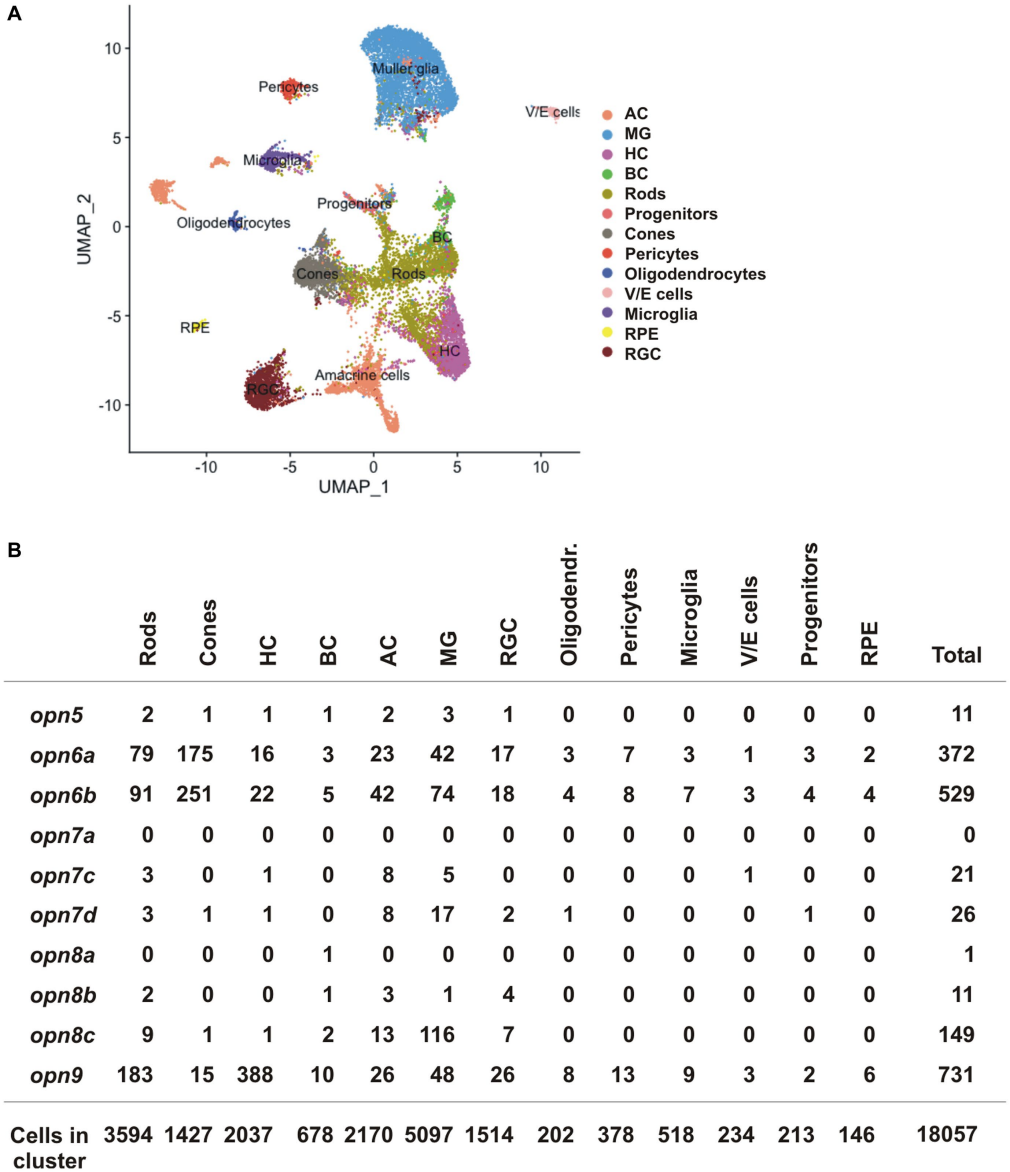
scRNA-seq data from the adult zebrafish (Hoang et al., 2020). **(A)** UMAP plot featuring annotated retinal cell types (Supplementary Table S1). **(B)** Quantification of cells which express *neuropsin* mRNA organized by cell type detected by scRNA-seq.

We next compared our data obtained in *X. laevis* to publicly available scRNA-seq datasets from the adult zebrafish, mouse, and chicken retina (Hoang et al., 2020). The datasets were generated recently to analyze gene expression of normal and injured retinas (Hoang et al., 2020). We isolated the scRNA-seq data of the control (uninjured) retina and analyzed the expression of neuropsins in different retinal cell-type clusters. *opn5* was the only neuropsin gene detected in the chicken and mouse dataset, however, almost no cells showed expression (5 *opn5^+^* AC and 1 *opn5^+^* RGC of 8,895 chicken retinal cells; no *opn5^+^* neuropsin gene of 14,891 mouse retinal cells). These results can be explained by a lack of evolutionary conservation of neuropsins, the small number of retinal cells in the chicken and mouse datasets, and low *opn5* mRNA levels. In contrast, the zebrafish dataset contained more retinal cells (18,057 cells), and several neuropsins were detected (Figures 7A,B). Of note, some of the retinal cluster annotations present in the original dataset, such as GABAergic and glycinergic ACs as well as activated and resting MG cells, were grouped as ACs and MG cells, respectively, in our analysis.

*opn5* was detected in only a handful of cells in the adult zebrafish retina (11 *opn5*^+^ cells/18,057 total cells), spread between different cell types (rods, cones, BC, HC, AC and MG cells) (Figure 7B). In contrast, other neuropsins were expressed in more cells and the data was supportive of our results in *X. laevis*: (i) *opn6a* and o*pn6b* were detected mainly in cones and rods [*opn6a*: 175/1427 cones (12.26%), 79/3,594 rods (2.2%); *opn6b*: 251/1,427 cones (17.6%); 91/3,594 rods (2.5%)]. Of note, zebrafish *opn6b* is homologous to *opn6a* generated during the teleost whole genome duplication and differs from the *opn6b* in *X. laevis*; (ii) the three *opn7* genes were barely expressed in the retina; and (iii) *opn8c* was expressed mainly in MG cells (116 cells/5,097 MG, 2.27%) and ACs (13/2,170 cells, 0.6%). Additionally, *opn9* that is present in teleosts but lost in tetrapods, was expressed in HCs (388/2037 cells, 19%), which agrees with findings from ISH performed in adult zebrafish (Davies et al., 2015).

## Discussion

Here, we studied the expression of the *neuropsin* family members in the *X. laevis* retina and compared our results with scRNA-seq data from zebrafish, chicken, and mouse. We demonstrated that: (1) *opn6a* and *opn6b* expression initiates at stage 35, at the same time as the classical opsins and melanopsin (Figure 1 and Table 1). *opn5* and *opn8* mRNA induction occurs several hours later (stage 37), (2) *opn6a* and *opn6b* are the neuropsins of newly born photoreceptors (Figure 2). The number of cells expressing *opn6a*/*b* diminished as eye circuity and lamination progresses, such that, by stage 43/44 (mature retina), the strongest expression is within newly born photoreceptors that border the CMZ (Figure 2). The *opn6* genes are also expressed in adult zebrafish in photoreceptors, but mRNA is not detected in chicken and mouse scRNA-seq data (Figure 7), (3) *opn5* mRNA is present both in the INL in a subpopulation of BCs and ACs, and in a small proportion of cells in the GCL (Figure 3). Similarly, the chicken scRNA-seq data point to *opn5* mRNA in ACs and RGCs (Figure 7), (4) *opn8* is expressed only by Pax6^+^ cells of the INL and the GCL (Figure 4), while in zebrafish the two clusters with highest expression are MG and ACs (Figure 7), (5) *opn8* shows little or no co-localization with either *opn5* or *opn4a*, while *opn5* shows moderate expression (~40%) in *opn4a* ^+^ RGCs (Figure 5), and (6) *opn5-* and *opn8*- expressing cells (>60%) induce *c-fos* expression and are part of retinal circuits that activate with a light stimulus (Figure 6).

Particularly interesting is the expression of *opn6a* and *opn6b* in what appears to be newly generated photoreceptors that emerge in the central retina near the end of embryogenesis, and subsequently are generated at the periphery of the mature retina by the progenitors of the CMZ. These data suggest that *opn6* functions in early photoreceptor differentiation, at the same time that expression of rod and cone opsins become pronounced (stage 35/36) (Chang and Harris, 1998). Expression subsequently refines to specific in mature and differentiated photoreceptors of the central retina (Figure 2), while less differentiated photoreceptors express both *opn6a* (rods; Rho^+^) and *opn6b* (cones; Calbindin^+^) mRNAs.

**Figure 8:**
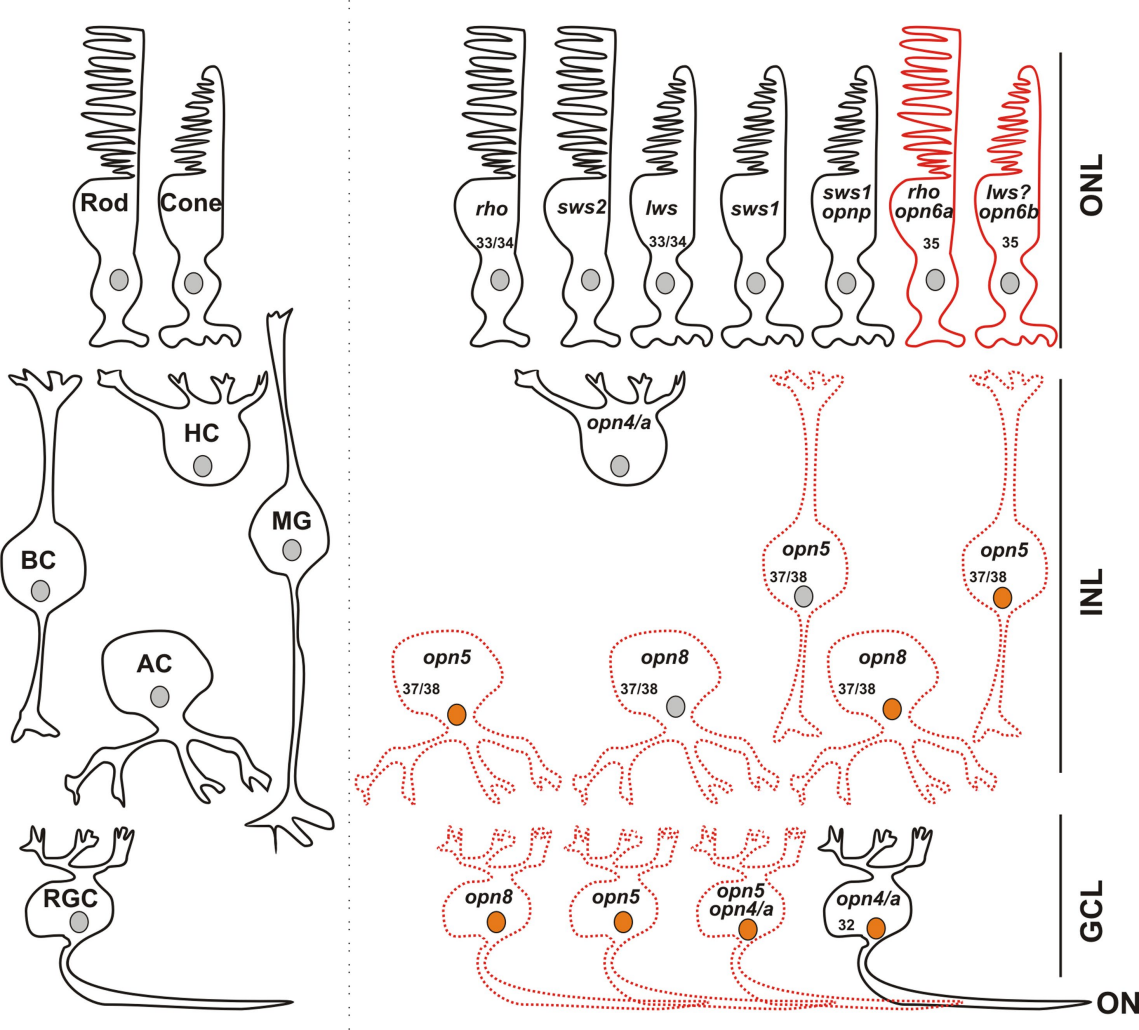
Opsin-expressing cells in the retina of a stage 43/44 *Xenopus* tadpole. Photoreceptors located in the outer nuclear layer (ONL) express the classical opsins in rods (*rho* or *sws2*; Kojima et al., 2017) and cones (*lws* and *sws1*). Pinopsin (*opnp*) is co-expressed in a few *sws1*^+^ cells (Bertolesi et al., 2021). Labeled with red are cells expressing *opn6a* and *opn6b* in rods (rho^+^) and cones, likely *lws^+^*, respectively. In the inner nuclear layer (INL), horizontal cells (HC) co-express the two melanopsin genes (*opn4* and *opn4a*), bipolar cells (BC) express *opn5*, while amacrine cells (AC) express *opn5* or *opn8*. Different cells in the ganglion cell layer (GCL) express opsins, including *opn5*, *opn8*, *opn4*/*opn4a*, and a few cells that contain *opn4*/*a* and *opn5*. Cells that express *c-fos* in response to exposure to white light are shown with an orange nucleus. Although classical photoreceptors are *c-fos* negative, they are highly likely activated by the white light exposure. We propose that opsin-expressing cells in the INL or GCL that exhibit light-induced *c-fos* expression serve as second- or third-order neurons, which receive synaptic inputs from photoreceptors or other retinal cells. We cannot rule out, however, that the opsin-expressing cells of the INL and GCL are intrinsically photosensitive and induce *c-fos* mRNA upon light stimulation, acting as first-order neurons. *opn4*/*a^+^* HCs do not express *c-fos* while *opn4*/*a^+^* RGCs do (Bertolesi et al., 2014). Opsin-expressing cells that we describe in this manuscript are either labeled with red solid lines (PR; photoreceptors), or red dotted lines as having the potential to be photosensitive in *Xenopus*, since direct light activation has not yet been demonstrated. Excluded are all the photoreceptors, and HCs and RGCs expressing melanopsins, for which photosensitivity was demonstrated in fish (Cheng et al., 2009) and mammals (Thoreson and Dacey, 2019). Numbers in the cells indicate the development stage of *Xenopus* when opsin expression is first detected (See also Table 1).

In combination with known photoreceptor opsins, *opn6a* and *opn6b* add complexity to the classical photoreceptor subtypes located in the *Xenopus* ONL, with seven different types now possible (Figure 8): (1) red rods, Rhodopsin (Rho)-expressing cells at ~47%–50% of the total photoreceptors, (2) green rods, atypical photoreceptors (2%–3%) found in amphibians that express the *short-wavelength-sensitive 2 opsin* (*sws2*), a blue-sensitive opsin located in cells with a rod-shaped structure (Gábriel and Wilhelm, 2001; Kojima et al., 2017), (3) cones that express the *long wavelength cone opsin* (*lws*; ~40% of photoreceptors), (4) cones that express the *short wavelength-sensitive 1 opsin* (*sws1*; ~10% of photoreceptors) (Starace and Knox, 1998; Bertolesi et al., 2021), (5) a small proportion of cones (<1%) that express *pinopsin* (*opnp*) alongside *sws1* (Bertolesi et al., 2021), (6) rods that co-express *rho* and *opn6a*, and (7) cones likely expressing *lws*, the most abundant Calbindin^+^ cell, and *opn6b*.

During evolution, *opn6* was maintained from teleosts to birds. *opn6* disappeared in the mammalian lineage (Davies et al., 2015; Beaudry et al., 2017), a feature that correlates with the loss of an adult retinal stem cell niche, the CMZ. The CMZ functions to add retinal neurons to the periphery of the neural retina in larval and adult fish and frogs (Fischer et al., 2013). Reptiles maintain a CMZ that differs among species and between embryonic and postnatal retina, and with a small proliferative capacity in the adult (Eymann et al., 2019). Proliferating progenitor-like cells are also present in the retinas of some birds, although located in a smaller structure than the CMZ detected in amphibians and fish, while mammals do not have a CMZ (Fischer et al., 2013). The presence of the neuroepithelium of the CMZ and the evolutionary conservation of *opn6* (expressed by photoreceptors abutting the CMZ) may be explained by the requirement for continued expression of *opn6* with ongoing production of new photoreceptors by the CMZ of the frog and fish retina over the lifetime.

The presence of *opn5* in cells located in the INL appears to decrease during evolution, though expression in RGCs is maintained. We find *opn5* mRNA is first detected at stage 37/38, is pronounced at stage 41, and sparse by stage 43, which agrees with our previous data showing minimal expression of *opn5* in the INL and RGC layer in a mature *X. laevis* retina (Bertolesi et al., 2021). At the peak of its expression at stage 41, *opn5* mRNA is distributed between BCs (~70%–75%), ACs (~10%) and RGCs (~20%). Similarly, in teleost fish, *opn5m* mRNA is present in the inner region of the INL and *opn5m2* mRNA in the outer edge of the INL (Sato et al., 2016). In mammals, however, *opn5* expression is restricted to RGCs (Calligaro et al., 2022; D’Souza et al., 2022), where *opn5* is used for photoentrainment (Buhr et al., 2015) and eye growth during development (Nguyen et al., 2019). *opn5m* mRNA is detected in RGCs, ACs, and MG within the avian retina during development and after hatching (Rios et al., 2019; Guido et al., 2020; Calligaro et al., 2022). Thus, *opn5* expression is present in INL cells of teleosts and amphibians, but is reduced in birds and absent in mammals.

We first detected *opn8* in the retina at stage 37/38 in a handful of ACs and RGCs, in a ratio of 7:3. The expression of the paralogues in zebrafish (*opn8a*, *8b*, and *8c*) differs somewhat. For example, the scRNA-seq data indicates that *opn8c* is most highly expressed in MGs, followed by ACs and photoreceptors, while *opn8a* and *opn8b* exhibit minimal expression in retinal cells. The laminar localization of *opn8* transcripts in the adult zebrafish retina support this cellular localization; *opn8a* and *opn8c* mRNAs are in the outer INL where BCs and HCs reside, *opn8b* and *opn8c* mRNAs are in the inner INL where the somas of ACs and MG are present, and *opn8b* mRNA is in the GCL (Davies et al., 2015). Thus, the *opn8* transcript in *X. laevis* appears to show a more restricted expression (ACs and RGCs) than its counterparts in zebrafish (potentially BCs, ACs, HCs, MG and RGCs). Evolutionarily, *opn8* genes are found in vertebrates from Holostei to birds, but are gone from mammals (Davies et al., 2015; Beaudry et al., 2017). The three *opn8* genes found in zebrafish also exist in the spotted gar (Beaudry et al., 2017), the common ancestor of Holostei and Teleostei. The evolutionary process of *opn8* in vertebrates is unknown, and the question of which fish *opn8* gene corresponds to the one conserved in tetrapods remains unanswered. What we do know is that the evolutionary rate of *opn8c* is higher than the other homologues (*opn8a* and *opn8b*) in the basal branch of teleost (Beaudry et al., 2017), which suggests an adaptative advantage. When zebrafish and *Xenopus opn8* are compared at the amino acid level, however, *opn8c* shows the least conservation (37% identity for *opn8c*, 42% for *opn8b*, and 56% for *opn8a*). Genetic and biochemical characterizations of *opn8* in these two model organisms may help us understand its physiology and evolution.

Our data suggests a lack of co-expression of neuropsins, which increases considerably the repertoire of potential photosensitive cells in the INL and GCL. We find that in the INL and the GCL, most cells express only one opsin. Indeed, *opn8^+^* ACs and RGCs do not express either *opn5* or *opn4*/*a* and only a modest proportion (<40%) of *opn5*^+^ cells co-express melanopsin (Figures 5, 8). Thus, in the GCL, potential photosensitive RGCs are *opn4^+^*, *opn5^+^* and *opn8^+^*, while in the INL the repertoire of photosensitive cells includes *opn5^+^* or *opn8^+^*ACs, *opn5^+^* BCs, and *opn4*/*a^+^* HCs (Figure 8). *opn5^+^* MG and *opn4^+^* HCs in birds and zebrafish, respectively, are known to be photosensitive (Cheng et al., 2009; Guido et al., 2020). This is also true of melanopsin-expressing RGCs in mammals (Thoreson and Dacey, 2019). Of note, the two *Xenopus* melanopsin genes, *opn4* and *opn4a,* are co-expressed in HCs and RGCs (Bertolesi et al., 2014). Interestingly, the *opn4a* gene was lost in mammals, but was positively selected for, through unknown evolutionary selection pressures, in other tetrapods and teleosts (Borges et al., 2012).

Co-expression of opsins in photoreceptors is more common, and would increase their range of wavelength sensitivity. For example, co-expression of a few cone opsins contributes to the UV-sensitivity of anemonefish (Mitchell et al., 2021), and tropical fish that live in clear waters with broad light environments express up to five opsin genes, thus increasing the gradient of visual sensitivity (Torres-Dowdall et al., 2021). In *Xenopus*, the four classical photoreceptors classified by their expression of *rho*, *sws2*, *sws1*, and *lws* are now increased to seven by the co-expression of *pinopsin* with *sws1* (Bertolesi et al., 2021), and *opn6a* and *opn6b* with *rho* and likely with *lws*, respectively. Understanding the photosensitive properties of these photoreceptors will require electrophysiological and/or biochemical studies to define the unknown spectral characteristics of *opn6a* and *opn6b*.

Our previous work suggested that with light stimulation, *c-fos* is not induced in first-order neurons of the retina, such as the photoreceptors that express *opn6a* and *opn6b,* but is in second-order and third-order neurons of the INL and GCL (Bertolesi et al., 2014). For the INL, some but not all opsin-expressing cells respond to white light activation with *c-fos* induction. For instance, *c-fos* is not induced in *opn4*/*a^+^-*expressing HCs (Bertolesi et al., 2014) that are found in amphibians (Provencio et al., 1998; Bertolesi et al., 2014), birds (Verra et al., 2011), and teleosts (Cheng et al., 2009), but not mammals. These cells exhibit intrinsic photosensitivity in fish (Cheng et al., 2009), act as photoreceptors in birds (Morera et al., 2016), and in amphibians we postulated that *opn4*/a^+^ HCs recognize light reflected from the substrate during background adaptation (Bertolesi and McFarlane, 2018). Interestingly, several *c-fos*^+^ cells located in the outer region of the INL, which are likely the classical HCs (Prox1^+^) that turn on c-fos when the eye receives light stimulation via synaptic inputs. Additionally, several ACs and BCs that express neuropsins are *c-fos*^+^ after white light illumination. This is true of 60% of the *opn5^+^* Otx2^+^ BCs. The observation that 40% of the cells do not turn on *c-fos* suggests that the *opn5^+^* Otx2^+^ BCs consist of both ON (Otx2^+^ Isl1^+^) and OFF (Otx2^+^) BCs (Bertolesi et al., 2014); the latter population participate in circuits that shut down with light stimulation. Similarly, only a subpopulation of the *opn8^+^* ACs show *c-fos* induction, suggesting that *opn8^+^* ACs participate in both ON and OFF circuits (Figure 6). In contrast, almost all neuropsin- and melanopsin- expressing cells in the GCL are activated upon light stimulation (Bertolesi et al., 2014) (Figures 6, 8).

This study gave a better understanding of retinal neuropsin-expressing cells, their developmental onset and activation by light. By using specific markers, we defined the cell types that express neuropsins. The expression of neuropsins in the ONL (*opn6a* and *opn6b*) increase the number of photoreceptors present in *Xenopus*, while expression in cells in the INL and GCL increase dramatically the number of potentially photosensitive cells. The onset of expression of *opn6a* and *opn6b* (stage 35) coincides with that of ‘classical’ opsins, and suggests a role in the differentiation and/or function of newborn photoreceptors. The onset of expression of *opn5* (BC, AC and RGC) and *opn8* (AC and RGC) occurs slightly later (stage 37) when retinal circuits become “wired” (stage 37/38). The fact that neuropsins are present as the retinal circuits are established argues for the importance of intrinsic photosensitivity within interneurons in modulating activity in the circuits, an interesting idea that needs exploring. Finally, by comparing neuropsin expression between different species we provide insight into evolutionary differences. Our data suggest that the numbers of different types of neuropsin-expressing cells in the INL and GCL likely diminished during evolution (teleost > amphibians > birds > mammals). In mammals, opsin-expressing cells are in photoreceptors and RGCs (*opn*4 and *opn*5) while the modulatory interneurons do not express opsins. Understanding the evolution of retinal circuits in vertebrates will be helped by a better grasp of neuropsin physiology.

## Data availability statement

The names of the repository/repositories and accession number(s) are mentioned in materials and methods section or can be found in the article/Supplementary material.

## Ethics statement

The animal study was approved by Animal Care and Use Committee at the University of Calgary. The study was conducted in accordance with the local legislation and institutional requirements.

## Author contributions

LM: Conceptualization, Data curation, Formal Analysis, Investigation, Methodology, Writing–original draft, Writing–review & editing. SS: Software, Data curation, Investigation, Methodology, Writing–original draft, Writing–review & editing. GB: Validation, Data curation, Investigation, Methodology, Writing–original draft, Writing–review & editing, Conceptualization, Formal Analysis, Supervision. SM: Conceptualization, Funding acquisition, Project administration, Supervision, Writing–original draft, Writing–review & editing.

## Funding

The author(s) declare financial support was received for the research, authorship, and/or publication of this article. This work was supported by a Discovery Grant from the Natural Sciences and Engineering Research Council of Canada (NSERC) to SM (PIN03909-2018). LM was a recipient of an NSERC summer studentship award.

## Conflict of interest

The authors declare that the research was conducted in the absence of any commercial or financial relationships that could be construed as a potential conflict of interest.

## Publisher’s note

All claims expressed in this article are solely those of the authors and do not necessarily represent those of their affiliated organizations, or those of the publisher, the editors and the reviewers. Any product that may be evaluated in this article, or claim that may be made by its manufacturer, is not guaranteed or endorsed by the publisher.

## Supplementary material

The Supplementary material for this article can be found online at: https://www.frontiersin.org/articles/10.3389/fncel.2023.1266945/full#supplementary-material

## Footnotes

^1^ www.xenbase.org/

## Glossary

BCIP: 5-Bromo-4-chloro-3-indolyl phosphate
CMZ: Ciliary marginal zone
DEPC: Diethylpyrocarbonate
DIG: Digoxigenin
D-FISH: Double fluorescent *in-situ* hybridization
FITC: Fluorescein isothiocyanate
GCL: Ganglion cell layer
INL: Inner nuclear layer
LWS: Long-wavelength sensitive
MOPS: 3-N-morpholino propanesulfonic acid
NBT: Nitro blue tetrazolium chloride
OCT: Optimal cutting temperature
ONL: Outer nuclear layer
RGC: Retinal ganglion cell
RT-PCR: Reverse-transcription polymerase chain reaction
SWS: Short-wavelength sensitive
TSA: Tyramide signal amplification
UV: Ultra-violet

## References

[ref1] Beaudry F. Iwanicki T. W. Mariluz B. Darnet S. Brinkmann H. Schneider P.. (2017). The non-visual opsins: eighteen in the ancestor of vertebrates, astonishing increase in ray-finned fish, and loss in amniotes. J. Exp. Zool. B 328, 685–696. doi: 10.1002/jez.b.22773 29059507

[ref2] Bertolesi G. E. Atkinson-Leadbeater K. Mackey E. M. Song Y. N. Heyne B. McFarlane S. (2020). The regulation of skin pigmentation in response to environmental light by pineal type II opsins and skin melanophore melatonin receptors. J. Photochem. Photobiol. B 212:112024. doi: 10.1016/j.jphotobiol.2020.112024 32957069

[ref3] Bertolesi G. E. Debnath N. Atkinson-Leadbeater K. Niedzwiecka A. McFarlane S. (2021). Distinct type II opsins in the eye decode light properties for background adaptation and behavioural background preference. Mol. Ecol. 30, 6659–6676. doi: 10.1111/mec.16203 34592025

[ref4] Bertolesi G. E. Hehr C. L. McFarlane S. (2014). Wiring the retinal circuits activated by light during early development. Neural Dev. 9:3. doi: 10.1186/1749-8104-9-3 24521229 PMC3937046

[ref5] Bertolesi G. E. McFarlane S. (2018). Seeing the light to change colour: an evolutionary perspective on the role of melanopsin in neuroendocrine circuits regulating light-mediated skin pigmentation. Pigment Cell Melanoma Res. 31, 354–373. doi: 10.1111/pcmr.12678 29239123

[ref6] Borges R. Johnson W. E. O’Brien S. J. Vasconcelos V. Antunes A. (2012). The role of gene duplication and unconstrained selective pressures in the melanopsin gene family evolution and vertebrate circadian rhythm regulation. PLoS One 7:e52413. doi: 10.1371/jour-nal.pone.0052413 23285031 PMC3528684

[ref7] Briggs J. A. Weinreb C. Wagner D. E. Megason S. Peshkin L. Kirschner M. W.. (2018). The dynamics of gene expression in vertebrate embryogenesis at single-cell resolution. Science 360, 1–23. doi: 10.1126/science.aar5780 PMC6038144 29700227

[ref8] Buhr E. D. Yue W. W. S. Ren X. Jiang Z. Liao H.-W. R. Mei X.. (2015). Neuropsin (*opn5*)-mediated photoentrainment of local circadian oscillators in mammalian retina and cornea. Proc. Natl. Acad. Sci. U. S. A. 112, 13093–13098. doi: 10.1073/pnas.1516259112 26392540 PMC4620855

[ref9] Calligaro H. Dkhissi-Benyahya O. Panda S. (2022). Ocular and extraocular roles of neuropsin in vertebrates. Trends Neurosci. 45, 200–211. doi: 10.1016/J.TINS.2021.11 34952723 PMC8854378

[ref10] Chang W. S. Harris W. A. (1998). Sequential genesis and determination of cone and rod photoreceptors in *Xenopus*. J. Neurobiol. 35, 227–244. 9622007

[ref11] Cheng N. Tsunenari T. King-Wai Y. (2009). Intrinsic light response of retinal horizontal cells of teleosts. Nature 460, 899–903. doi: 10.1038/nature08175 19633653 PMC2737592

[ref12] D’Souza S. P. Swygart D. I. Wienbar S. R. Upton B. A. Zhang K. X. Mackin R. D.. (2022). Retinal patterns and the cellular repertoire of neuropsin (*opn5*) retinal ganglion cells. J. Comp. Neurol. 530, 1247–1262. doi: 10.1002/CNE.25272 34743323 PMC8969148

[ref13] Davies W. I. L. Tamai T. K. Zheng L. Fu J. K. Rihel J. Foster R. G.. (2015). An extended family of novel vertebrate photopigments is widely expressed and displays a diversity of function. Genome Res. 25, 1666–1679. doi: 10.1101/gr.189886.115 26450929 PMC4617963

[ref14] Díaz N. M. Morera L. P. Guido M. E. (2016). Melanopsin and the non-visual photochemistry in the inner retina of vertebrates. Photochem. Photobiol. 92, 29–44. doi: 10.1111/php.12545 26500165

[ref15] Eymann J. Salomies L. Macrì S. (2019). Variations in the proliferative activity of the peripheral retina correlate with postnatal ocular growth in squamate reptiles. J. Comp. Neurol. 527, 2356–2370. doi: 10.1002/cne.24677 30860599 PMC6766921

[ref16] Fischer A. J. Bosse J. L. El-hodiri H. M. (2013). The ciliary marginal zone (CMZ) in development and regeneration of the vertebrate eye. Exp. Eye Res. 116, 199–204. doi: 10.1016/j.exer.2013.08.018 24025744

[ref17] Frederick J. M. Rayborn M. E. Hollyfield J. G. (1989). Serotoninergic neurons in the retina of *Xenopus laevis*: selective staining, identification, development, and content. J. Comp. Neurol. 281, 516–531. doi: 10.1002/cne.902810403 2708578

[ref18] Gábriel R. Wilhelm M. (2001). Structure and function of photoreceptor and second-order cell mosaics in the retina of *Xenopus*. Int. Rev. Cytol. 210, 77–120. doi: 10.1016/s0074-7696(01)10004-5 11580209

[ref19] Guido M. E. Marchese N. A. Rios M. N. Morera L. P. Diaz N. M. Garbarino-Pico E.. (2020). Non-visual opsins and novel photo-detectors in the vertebrate inner retina mediate light responses within the blue spectrum region. Cell. Mol. Neurobiol. 42, 59–83. doi: 10.1007/s10571-020-00997-x 33231827 PMC11441211

[ref20] Hirsch N. Harris W. A. (1997). *Xenopus* Pax-6 and retinal development. J. Neurobiol. 32, 45–61. 8989662

[ref21] Hoang T. Wang J. Boyd P. Wang F. Santiago C. Yoo S.. (2020). Gene regulatory networks controlling vertebrate retinal regeneration. Science 370, 1–34. doi: 10.1126/science.abb8598.Gene PMC7899183 33004674

[ref22] Hollyfield J. G. Rayborn M. E. Sarthy P. V. Lam D. M. K. (1980). Retinal development: time and order of appearance of specific neuronal properties. Neurochem. Int. 1, 93–101. doi: 10.1016/0197-0186(80)90053-4 20487728

[ref23] Holt C. E. (1989). A single-cell analysis of early retinal ganglion cell differentiation *Xenopus*: from soma to axon tip. J. Neurosci. 9, 3123–3145. doi: 10.1523/JNEUROSCI.09-09-03123.1989 2795157 PMC6569658

[ref24] Holt C. E. Bertsch T. W. Ellis H. M. Harris W. A. (1988). Cellular determination in the *Xenopus* retina is independent of lineage and birth date. Neuron 1, 15–26. doi: 10.1016/0896-6273(88)90205-x 3272153

[ref25] Huerta J. J. Llamosas M. M. Cernuda-Cernuda R. García-Fernández J. M. (1997). Fos expression in the retina of *rd*/*rd* mice during the light/dark cycle. Neurosci. Lett. 232, 143–146. doi: 10.1016/s0304-3940(97)00595-8 9310300

[ref26] Kojima K. Matsutani Y. Yamashita T. Yanagawa M. Imamoto Y. Yamano Y.. (2017). Adaptation of cone pigments found in green rods for scotopic vision through a single amino acid mutation. Proc. Natl. Acad. Sci. U.S.A. 114, 5437–5442. doi: 10.1073/pnas.1620010114 28484015 PMC5448186

[ref27] Lamb T. D. (2013). Evolution of phototransduction, vertebrate photoreceptors and retina. Prog. Retin. Eye Res. 36, 52–119. doi: 10.1016/j.preteyeres.2013.06.001 23792002

[ref28] Liu Z. Hamodi A. S. Pratt K. G. (2016). Early development and function of the *Xenopus* tadpole retinotectal circuit. Curr. Opin. Neurobiol. 41, 17–23. doi: 10.1016/J.CONB.2016.07.002 27475307

[ref29] Mitchell L. J. Cheney K. L. Cortesi F. Lu M. Marshall J. Michie K. (2021). Molecular evolution of ultraviolet visual opsins and spectral tuning of photoreceptors in anemonefishes (*Amphiprioninae*). Genome Biol. Evol. 13, 1–14. doi: 10.1093/gbe/evab184 PMC8511661 34375382

[ref30] Morera L. P. Díaz N. M. Guido M. E. (2016). Horizontal cells expressing melanopsin x are novel photoreceptors in the avian inner retina. Proc. Natl. Acad. Sci. U.S.A. 113, 13215–13220. doi: 10.1073/pnas.1608901113 27789727 PMC5135307

[ref31] Nguyen M. T. Vemaraju S. Nayak G. Odaka Y. Buhr E. D. Alonzo N.. (2019). An opsin 5-dopamine pathway mediates light-dependent vascular development in the eye. Nat. Cell Biol. 21, 420–429. doi: 10.1038/s41556-019-0301-x 30936473 PMC6573021

[ref32] Perron M. Harris W. A. (1999). “Cellular determination in amphibian retina” in Cell lineage and fate determination. ed. Moody S. A.. 1st ed (New York: Academic Press), 353–368.

[ref33] Provencio I. Jiang G. De Grip W. J. Hayes W. P. Rollag M. D. (1998). Melanopsin: an opsin in melanophores, brain, and eye. Proc. Natl. Acad. Sci. U. S. A. 95, 340–345. doi: 10.1073/pnas.95.1.340 9419377 PMC18217

[ref34] Rios M. N. Marchese N. A. Guido M. E. (2019). Expression of non-visual opsins *opn3* and *opn5* in the developing inner retinal cells of birds. Light-responses in Müller glial cells. Front. Cell. Neurosci. 13:376. doi: 10.3389/fncel.2019.00376 31474836 PMC6706981

[ref35] Sato K. Yamashita T. Haruki Y. Ohuchi H. Kinoshita M. (2016). Two UV-sensitive photoreceptor proteins, *opn5m* and *opn5m2* in ray-finned fish with distinct molecular properties and broad distribution in the retina and brain. PLoS One 11:e0155339. doi: 10.1371/journal.pone.0155339 27167972 PMC4864311

[ref36] Session A. M. Uno Y. Kwon T. Chapman J. A. Toyoda A. Takahashi S.. (2016). Genome evolution in the allotetraploid frog *Xenopus laevis*. Nature 538, 336–343. doi: 10.1038/nature19840 27762356 PMC5313049

[ref37] Sonam S. Bangru S. Perry K. J. Chembazhi U. V. Kalsotra A. Henry J. J. (2022). Cellular and molecular profiles of larval and adult *Xenopus* corneal epithelia resolved at the single-cell level. Dev. Biol. 491, 13–30. doi: 10.1016/j.ydbio.2022.08.007 36049533 PMC10241109

[ref38] Starace D. M. Knox B. E. (1998). Cloning and expression of a *Xenopus* short wavelength cone pigment. Exp. Eye Res. 67, 209–220. doi: 10.1006/exer.1998.0507 9733587

[ref39] Szaro B. G. Gainer H. (1988). Immunocytochemical identification of non-neuronal intermediate filament proteins in the developing *Xenopus laevis* nervous system. Dev. Brain Res. 43, 207–224. doi: 10.1016/0165-3806(88)90100-9 2460198

[ref40] Takahashi Y. Hisatomi O. Sakakibara S. Tokunaga F. Tsukahara Y. (2001). Distribution of blue-sensitive photoreceptors in amphibian retinas. FEBS Lett. 501, 151–155. doi: 10.1016/S0014-5793(01)02632-1 11470275

[ref41] Thoreson W. B. Dacey D. M. (2019). Diverse cell types, circuits, and mechanisms for color vision in the vertebrate retina. Physiol. Rev. 99, 1527–1573. doi: 10.1152/physrev.00027.2018 31140374 PMC6689740

[ref42] Torres-Dowdall J. Karagic N. Härer A. Meyer A. (2021). Diversity in visual sensitivity across Neotropical cichlid fishes via differential expression and intraretinal variation of opsin genes. Mol. Ecol. 30, 1880–1891. doi: 10.1111/mec.15855 33619757

[ref43] Verra D. M. Contin M. A. Hicks D. Guido M. E. (2011). Early onset and differential temporospatial expression of melanopsin isoforms in the developing chicken retina. Invest. Ophthalmol. Vis. Sci. 52, 5111–5120. doi: 10.1167/iovs.11-75301 21676907

[ref44] Viczian A. S. Vignali R. Zuber M. E. Barsacchi G. Harris W. A. (2003). XOtx5b and XOtx2 regulate photoreceptor and bipolar fates in the *Xenopus* retina. Development 130, 1281–1294. doi: 10.1242/dev.00343 12588845

[ref45] Yamashita T. Ohuchi H. Tomonari S. Ikeda K. Sakai K. Shichida Y. (2010). *opn5* is a UV-sensitive bistable pigment that couples with Gi subtype of G protein. Proc. Natl. Acad. Sci. U. S. A. 107, 22084–22089. doi: 10.1073/pnas.1012498107 21135214 PMC3009823

[ref46] Yoshida K. Imaki J. Hitoshi F. Harada T. Ohki K. Matsuda H.. (1996). Differential distribution of CaM kinases and induction of *c-fos* expression by flashing and sustained light in rat retinal cells. Invest. Ophthalmol. Vis. Sci. 37, 174–179. 8550320

[ref47] Yu M. C. Li W. W. Liu K. Yew D. T. (1994). An immunohistochemical study of the *c-fos* protooncogene in the developing human retina. Neuroscience 60, 983–987. doi: 10.1016/0306-4522(94)90277-1 7936216

