# Supplementary material for: Cell-type expression and activation by light of neuropsins in the developing and mature Xenopus retina
Source: Front Cell Neurosci. 2023 Sep 20;17:1266945. doi: 10.3389/fncel.2023.1266945 (PMC10547888; doi:10.3389/fncel.2023.1266945)
Supplement: Supplementary file 1 [file Table_1.DOCX]

**Supplementary Table 1: Cell type annotation**

| Gene.ID | Gene.symbol | Marked.cell.type |
| --- | --- | --- |
| ENSDARG00000012667 | tfap2b | AC |
| ENSDARG00000055158 | prox1a | AC |
| ENSDARG00000041062 | calb2a | AC |
| ENSDARG00000036344 | calb2b | AC |
| ENSDARG00000014420 | elavl3 | AC |
| ENSDARG00000053130 | pcp4a | AC, BC |
| ENSDARG00000087386 | pcp4b | AC, BC |
| ENSDARG00000088810 | prox1b | AC, BC, HC, MG |
| ENSDARG00000004023 | isl1 | AC, BC, RGC |
| ENSDARG00000093411 | gad1a | AC, HC |
| ENSDARG00000027419 | gad1b | AC, HC |
| ENSDARG00000103379 | pax6a | AC, MG |
| ENSDARG00000045936 | pax6b | AC, MG |
| ENSDARG00000045639 | elavl4 | AC, RGC |
| ENSDARG00000028148 | pax2a | Astrocytes |
| ENSDARG00000032578 | pax2b | Astrocytes |
| ENSDARG00000018643 | igf2a | Astrocytes |
| ENSDARG00000033307 | igf2b | Astrocytes |
| ENSDARG00000057598 | s100b | Astrocytes |
| ENSDARG00000070494 | pdgfra | Astrocytes |
| ENSDARG00000025301 | gfap | Astrocytes, MG |
| ENSDARG00000104431 | slc1a3a | Astrocytes, MG |
| ENSDARG00000043148 | slc1a3b | Astrocytes, MG |
| ENSDARG00000037588 | bhlhe23 | BC |
| ENSDARG00000002576 | cabp5a | BC |
| ENSDARG00000028485 | cabp5b | BC |
| ENSDARG00000005574 | vsx2 | BC |
| ENSDARG00000068989 | gabra1 | Cone BC |
| ENSDARG00000069368 | nrn1la | Cone BC |
| ENSDARG00000061149 | nrn1lb | Cone BC |
| ENSDARG00000097008 | opn1mw1 | Cones |
| ENSDARG00000044280 | opn1mw2 | Cones |
| ENSDARG00000044279 | opn1mw3 | Cones |
| ENSDARG00000000638 | opn1mw4 | Cones |
| ENSDARG00000045677 | opn1sw1 | Cones |
| ENSDARG00000017274 | opn1sw2 | Cones |
| ENSDARG00000056511 | arr3a | Cones |
| ENSDARG00000021163 | thrb | Cones |
| ENSDARG00000098475 | arr3b | Cones |
| ENSDARG00000012297 | cnga3b | Cones |
| ENSDARG00000070726 | cnga3a | Cones |
| ENSDARG00000042529 | gnat2 | Cones, Cone BC |
| ENSDARG00000015537 | gad2 | GABAergic AC |
| ENSDARG00000045944 | slc6a1a | GABAergic AC |
| ENSDARG00000039647 | slc6a1b | GABAergic AC |
| ENSDARG00000059775 | slc32a1 | GABAergic AC |
| ENSDARG00000010878 | cdkn1ca | GABAergic AC |
| ENSDARG00000104903 | cdkn1cb | GABAergic AC |
| ENSDARG00000018534 | slc6a9 | Glycinergic AC |
| ENSDARG00000067964 | slc6a5 | Glycinergic AC |
| ENSDARG00000014018 | lhx1a | HC |
| ENSDARG00000061240 | cbln4 | HC |
| ENSDARG00000031598 | calb1 | HC |
| ENSDARG00000090387 | onecut2 | HC |
| ENSDARG00000007944 | lhx1b | HC |
| ENSDARG00000007982 | onecut1 | HC |
| ENSDARG00000057568 | nefla | HC, RGC |
| ENSDARG00000012426 | neflb | HC, RGC |
| ENSDARG00000021351 | nefma | HC, RGC |
| ENSDARG00000043697 | nefmb | HC, RGC |
| ENSDARG00000012504 | rlbp1a | MG |
| ENSDARG00000045808 | rlbp1b | MG |
| ENSDARG00000099776 | glula | MG |
| ENSDARG00000100003 | glulb | MG |
| ENSDARG00000013227 | glulc | MG |
| ENSDARG00000102004 | apoea | MG |
| ENSDARG00000040295 | apoeb | MG |
| ENSDARG00000006514 | her6 | MG |
| ENSDARG00000103554 | notch1a | MG |
| ENSDARG00000052094 | notch1b | MG |
| ENSDARG00000010565 | aqp4 | MG |
| ENSDARG00000043511 | prdx6 | MG |
| ENSDARG00000010008 | vim | MG |
| ENSDARG00000090815 | kcnj10a | MG |
| ENSDARG00000010434 | clu | MG, Astrocytes |
| ENSDARG00000071437 | ptprc | Microglia |
| ENSDARG00000076586 | csf2rb | Microglia |
| ENSDARG00000068036 | tmem119b | Microglia |
| ENSDARG00000095259 | tmem119a | Microglia |
| ENSDARG00000069552 | atoh7 | NeurogenicRPCs |
| ENSDARG00000020298 | btg2 | NeurogenicRPCs |
| ENSDARG00000043581 | gadd45aa | NeurogenicRPCs |
| ENSDARG00000104571 | gadd45ab | NeurogenicRPCs |
| ENSDARG00000019417 | gadd45ga | NeurogenicRPCs |
| ENSDARG00000010591 | foxn4 | NeurogenicRPCs |
| ENSDARG00000069139 | grik1a | OFF cone BC |
| ENSDARG00000040627 | grik1b | OFF cone BC |
| ENSDARG00000061000 | klhdc8a | OFF cone BC |
| ENSDARG00000040948 | olig1 | Oligodendrocytes |
| ENSDARG00000040946 | olig2 | Oligodendrocytes |
| ENSDARG00000036186 | mbpa | Oligodendrocytes |
| ENSDARG00000089413 | mbpb | Oligodendrocytes |
| ENSDARG00000037954 | tnnt1 | ON cone BC |
| ENSDARG00000058732 | scgn | ON cone BC |
| ENSDARG00000017742 | grm6a | ON cone BC |
| ENSDARG00000025671 | grm6b | ON cone BC |
| ENSDARG00000045589 | kcnj8 | Pericytes |
| ENSDARG00000045180 | acta2 | Pericytes |
| ENSDARG00000100897 | pdgfrb | Pericytes |
| ENSDARG00000051748 | ccnd2a | PrimaryPRCs |
| ENSDARG00000070408 | ccnd2b | PrimaryPRCs |
| ENSDARG00000089368 | hopx | PrimaryPRCs, BC, MG, Cone |
| ENSDARG00000031222 | lhx2b | PrimaryPRCs, MG |
| ENSDARG00000037964 | lhx2a | PrimaryPRCs, MG |
| ENSDARG00000011166 | cahz | PrimaryPRCs, MG |
| ENSDARG00000034423 | sncga | RGC |
| ENSDARG00000098380 | sncgb | RGC |
| ENSDARG00000035018 | thy1 | RGC |
| ENSDARG00000100244 | ebf3a | RGC |
| ENSDARG00000010083 | rbfox3a | RGC |
| ENSDARG00000074310 | rbfox3b | RGC |
| ENSDARG00000003971 | isl2a | RGC |
| ENSDARG00000053499 | isl2b | RGC |
| ENSDARG00000005559 | pou4f1 | RGC |
| ENSDARG00000069737 | pou4f2 | RGC |
| ENSDARG00000006206 | pou4f3 | RGC |
| ENSDARG00000099396 | rbpms | RGC |
| ENSDARG00000091287 | ebf3b | RGC, AC |
| ENSDARG00000042526 | sebox | Rod BC |
| ENSDARG00000039241 | prkca | Rod BC |
| ENSDARG00000019566 | neurod1 | Rod progenitors |
| ENSDARG00000099572 | hmgn2 | Rod progenitors |
| ENSDARG00000002193 | rho | Rods |
| ENSDARG00000100466 | nrl | Rods |
| ENSDARG00000011235 | otx2 | Rods |
| ENSDARG00000011989 | crx | Rods |
| ENSDARG00000013393 | guca1b | Rods |
| ENSDARG00000019752 | rom1a | Rods |
| ENSDARG00000026926 | rom1b | Rods |
| ENSDARG00000044199 | gnat1 | Rods |
| ENSDARG00000045904 | nr2e3 | Rods, Rodprogenitors |
| ENSDARG00000087937 | cdk4 | RPCs |
| ENSDARG00000101637 | ccnd1 | RPCs, MG |
| ENSDARG00000007480 | rpe65a | RPE |
| ENSDARG00000094752 | rpe65b | RPE |
| ENSDARG00000054420 | rpe65c | RPE |
| ENSDARG00000091298 | pmela | RPE |
| ENSDARG00000033760 | pmelb | RPE |
| ENSDARG00000078331 | best1 | RPE |
| ENSDARG00000037278 | lrata | RPE |
| ENSDARG00000096594 | lratb | RPE |
| ENSDARG00000039077 | tyr | RPE |
| ENSDARG00000060263 | pecam1 | V/E cells |
| ENSDARG00000075549 | cdh5 | V/E cells |
| ENSDARG00000004105 | tie1 | V/E cells |
| ENSDARG00000028663 | tek | V/E cells |
