# Supplementary figures and images for: Cell-type expression and activation by light of neuropsins in the developing and mature Xenopus retina
Source: Front Cell Neurosci. 2023 Sep 20;17:1266945. doi: 10.3389/fncel.2023.1266945 (PMC10547888; doi:10.3389/fncel.2023.1266945)

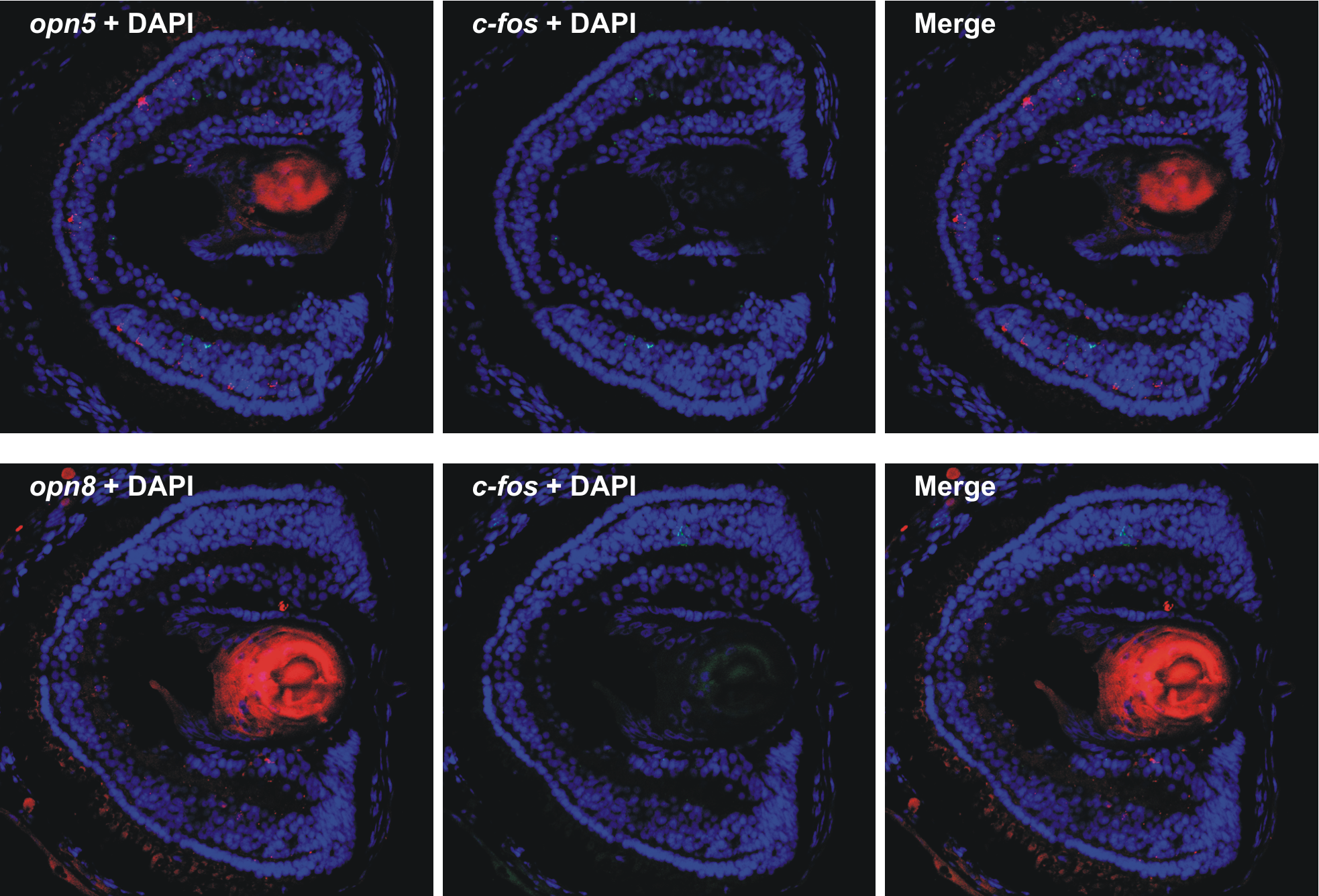

Supplement: Supplementary file 2 [file Image_1.TIF]
